# Experimental, numerical, and DIC analysis of high-performance VPP composites with multilayer glass fiber reinforcement

**DOI:** 10.1038/s41598-025-25027-y

**Published:** 2025-11-20

**Authors:** Ariyana Dwiputra Nugraha, Ferry Setiawan, Maulana Zulfa Khotami, Gesang Nugroho, Ardi Jati Nugroho Putro, Almas Aprilana, Alvin Dio Nugroho, Yi Chieh Wu, Farid Triawan, Murni Handayani, Yosephin Dewiani Rahmayanti, Muhammad Akhsin Muflikhun

**Affiliations:** 1 PLN Research Institute, Jakarta, Indonesia; 2 Department of Mechanical and Industrial Engineering, Universitas Gadjah Mada, Yogyakarta, Indonesia; 3 Department of Mechanical Engineering, Sekolah Tinggi Teknologi Kedirgantaraan, Yogyakarta, Indonesia; 4 Mechanical Engineering Department, Vytautas Magnus University, Kaunas, Lithuania; 5 Interdisiplinary Artificial Intelligence Center, National Chengchi University NCCU), Taipei, Taiwan; 6 Department of Mechanical Engineering, Sampoerna University, Jakarta, Indonesia; 7 Research Center for Nanotechnology Systems, National Research and Innovation Agency (BRIN), Puspiptek Area, Tangerang Selatan, Indonesia; 8 Center for Energy Studies, Universitas Gadjah Mada, Yogyakarta, Indonesia

**Keywords:** Aerospace engineering, Mechanical engineering

## Abstract

3D printing technology, particularly Vat Photopolymerization (VPP), which includes Digital Light Processing (DLP), has the advantage of producing precise products with good layer quality, but it has a weakness in low mechanical strength. This study aims to improve the mechanical characteristics of composites using standard resin reinforced with glass fiber in variations of 0, 1, 2, 3, and 4 layers. The resulting glass fiber-VPP composite specimens were analyzed through tensile test, flexural test, hardness test, and density test in accordance with ASTM standards. Validation of the experimental results was carried out using FEA (finite element analysis) and DIC (digital image correlation) methods, while evaluation of fracture microstructure phenomena was conducted using SEM testing. The results of this study showed that tensile strength increased by nearly three times. Specimens without reinforcement had an average ultimate tensile strength (UTS) of 20.1 MPa, while specimens with 4 layers of glass fiber had an average UTS of 59.3 MPa. As the number of glass fiber layers increased, the flexural strength of the composite tended to decrease, with the highest flexural strength observed in the specimen with 0 layers of glass fiber at 28.52 MPa, and the lowest in the specimen with 4 layers at 17.58 MPa. The addition of glass fiber led to an increase in density, reaching 1.25 g/cm^3^ in the specimen with 4 layers of glass fiber. Meanwhile, the hardness value decreased from 61.16 HD in the specimen with 0 layers to 47 HD in the specimen with 4 layers. The FEA and DIC simulations were consistent with the experimental data, supporting the reliability of the research results. This technology can be further applied in real-world applications, particularly in the biomedical, automotive, and aerospace industries.

## Introduction

Additive manufacturing, commonly referred to as three-dimensional (3D) printing, is a digital fabrication technique that forms products from a geometric model by gradually adding material, layer by layer, to produce precise three-dimensional geometry according to the design^1,2^. Types of 3D printing include selective laser sintering, inkjet printing, Vat Photopolymerization (VPP) which includes DLP (Digital Light Processing), and fused deposition modeling (FDM), where this technology is suitable for various fields such as medicine, aerospace technology, electronics and various other fields that require geometric accuracy in the product results^3,4^. Additive manufacturing (AM) of fiber composites is increasingly emerging as a key technology to produce complex structures and customizable mechanical properties, thus providing high design freedom^1,5^.

VPP has undergone significant development and made a great impact in the world of manufacturing, especially in making three-dimensional (3D) products with high efficiency and precision^6,7^. The advantage of VPP lies in its superior precision and layer quality compared to other methods such as Selective Laser Sintering (SLS) and Fused Deposition Modeling (FDM). Comparing three popular 3D printing methods, namely VPP (DLP), FDM, and Selective Laser Sintering (SLS), has shown that VPP excels in terms of precision and surface quality with a manufacturing process with relatively faster production time and lower cost, so additive manufacturing innovation can support sustainable manufacturing processes if designed with a systems approach and appropriate sustainable design methods^8–10^.

VPP uses a liquid photopolymer resin that hardens under ultraviolet (UV) light, this manufacturing enables printing with very high precision and layer quality, resulting in products with very high feature resolution, even down to the nano size^11^, For example 3D pyramid and hourglass micro lattice samples can be generated by micro-stereolithography^12^. Rapid and accurate manufacturing of products using VPP manufacturing methods has shown success in medical device development, improving cost efficiency and production effectiveness. These advantages make VPP highly desirable in applications that require high accuracy, such as in the automotive and healthcare industries^13–15^. One of the main challenges in the application of VPP-based materials is the low mechanical strength of pure polymers, which limits their use in components that require high durability and mechanical strength. materials produced through VPP techniques often have lower mechanical strength compared to other molding methods, such as injection molding^16^and FDM-filament winding^17^. The combination of standard and flexible resins can determine tensile strength, elongation, and hardness values, making it necessary to find the right composition to achieve a balanced strength and flexibility^18^. This is of particular concern in the automotive industry, where components such as car bumpers and UAV propellers require a combination of high precision and strength to meet stringent safety standards^19^. The ability of additive manufacturing (AM) technology to create fiber-reinforced composite materials has enabled the production of parts with a high degree of customization as well as much better mechanical properties compared to polymers without reinforcement^20^.

Research by Al-Qarni and Gad showed that some VPP-molded resins did not meet the flexural strength standards set by the International Organization for Standardization (ISO), indicating variability in the mechanical strength of the molded materials^21^. While Li revealed that the printing orientation can affect the mechanical properties of VPP materials with a difference of up to 25.95% in tensile strength, this suggests that a better understanding of the mechanical behavior of materials is essential for practical applications^22^. Research by Vidakis et al. showed that the use of nanocomposites in the VPP process can improve mechanical response compared to virgin materials^23^. Alshihabi showed that the use of nanoparticles such as Titanium Nitride and Boron Nitride in VPP resins can improve mechanical and thermal properties, making it highly relevant for applications in the automotive and aerospace sectors^19^. The addition of nano-graphite fabricated using the VPP method is also known to affect the strength, deformation, and crack resistance of the composite^24^. In addition, Shinde et al. emphasized the importance of developing self-healing composites, which shows the potential to improve the durability of parts molded with VPP^25^.

In addition to nanoparticle reinforcement, research with fiber reinforcement has also been conducted^26^. 3D printing using FDM reinforced with GFRP has shown an increase in tensile strength of more than two times, reaching 4977.3 N^27^. However, VPP 3D printing reinforced with GF has not been widely reported. Moumen et al. showed that the development of VPP resin-based composites with reinforcing materials such as glass fibers can improve the mechanical properties of the material without sacrificing the precision that characterizes this method By using glass fibers, these composites not only improve mechanical strength but also maintain high molding quality, thus making them an attractive option for applications that are more demanding of high mechanical strength^28^.

Additive manufacturing using the VPP method combined with reinforcement from glass fiber woven fabric is employed to produce composites with enhanced mechanical properties. This approach is more efficient and offers shorter production times compared to traditional methods such as vacuum infusion or hand lay-up, which require extended curing periods. The fabricated composites underwent flexural, impact, and interlaminar shear strength (ILSS) testing with variations in fiber orientation and volume fraction. The results demonstrated a flexural strength of up to 295 MPa, impact strength of 174 kJ/m^2^, and ILSS of 20.58 MPa. Glass fiber has proven effective as a reinforcing material in photosensitive resin for VPP based 3D printing. When modified with the silane coupling agent KH570, the interfacial bonding between the glass fiber and resin matrix was significantly enhanced. The composite reinforced with modified glass fiber (GF-KH570) achieved a tensile strength of up to 74.5 MPa, representing a 67% increase compared to pure resin and a 16% improvement over composites with unmodified glass fiber. Additionally, mechanical anisotropy was reduced to 4.2%, approaching isotropic behavior^29^. These findings highlight the high potential of modified glass fiber as a reinforcing agent for high-precision structural 3D printing applications.

The study by Song et al., evaluated the enhancement of mechanical properties in VPP-based photosensitive resin composites reinforced with glass fibers. The test results showed that the addition of glass fibers increased tensile strength by up to 50% and flexural strength by up to 143%, especially when the fibers were treated with silane. Additionally, the fiber coating method with a three-dimensional orthogonal structure improved tensile strength by up to 110% and flexural strength by up to 147%, compared to the continuous long fiber coating method. These findings confirm that surface treatment and fiber orientation play a crucial role in enhancing the mechanical performance of VPP composites. Therefore, further studies are needed to investigate the tensile and flexural properties of VPP-based photosensitive resin composites reinforced with glass fibers without the addition of silane as a coupling agent^30^.

Sano et al. conducted research on the effect of reinforcement of VPP resin-based composites using discontinuous glass powder and continuous glass fiber. The results showed that the tensile strength increased 2.1 times with glass powder reinforcement and 7.2 times with continuous glass fiber. However, in the use of short glass fiber reinforcement, it is reported that the uneven distribution of fibers causes voids, thus reducing the strength of the material^31^. VPP composites with photosensitive resin matrix and woven carbon fiber reinforcement with hybrid laminate variation showed an increase in tensile strength up to 4.2 times and flexural strength up to 10.7 times compared to pure VPP resin. It was also noted that the grooved texture structure of the carbon fiber reinforced 3D printed object had an effect on improving adhesion and load spreading^32,33^.

From previous research studies, it has been recognized the great potential in the development of VPP-based composites, on the other hand, there is a need in the industrial world for materials with a high level of precision, fast manufacturing processes and large mechanical strength, this makes research with VPP-based materials interesting to develop. Research conducted by Sano et al. demonstrated that glass fiber plain woven fabric can significantly enhance tensile strength. However, several weaknesses were identified, such as poor interfacial bonding between the fiber and the resin, the presence of micro-voids between the interfacial layers, and the use of only a single layer of reinforcing fiber. In the present study, an innovative development will be carried out by applying variations in multilayer glass fiber reinforcement and modifying the plain structure of the fiberglass woven fabric. This aims to improve the interfacial bonding strength between the matrix and the reinforcement^31^. This study aims to fill the gap in understanding the variation in the number of glass fiber (GF) reinforcement layers and their impact on mechanical properties such as flexural strength, hardness, and density. The novelty of this research lies in the innovation of developing composite materials using an VPP resin matrix reinforced with woven glass fiber, which has been modified in various layer configurations. In addition, this study also validates the experimental results using a combination of Finite Element Analysis (FEA) simulation and Digital Image Correlation (DIC) measurement method for deformation analysis.

## Materials and methods

### Materials selection and experimental setup

This study used eSUN Standard resin as the main material, which had a viscosity of 170–200 mPa·s, a density of 1.08–1.13 g/cm^3^, a tensile strength of 46–67 MPa, and a flexural strength of 46–72 MPa. eSUN Standard Resin is composed of 40–50% polyurethane acrylate, 20–40% monomer, 3–5% photoinitiator, and 2–5% pigment. Designed for precise 3D printing, it minimizes shrinkage during photocuring. Upon UV exposure, photoinitiators produce free radicals that trigger chain polymerization, solidifying the resin layer by layer into high-resolution prints^34^. As reinforcement, Woven Glass Fiber (GF) was used, with a density of 1.64017 g/cm^3^, areal density of 177.48 g/m^2^, and a consolidated thickness of 0.2 mm. The research was conducted using various state-of-the-art equipment, including the Anycubic Photon Ultra VPP 3D Printer for the printing process, as well as Anycubic’s Wash and Cure Machine for the post-printing stage to clean and harden the specimens, ensuring optimal results.

**Fig. 1:**
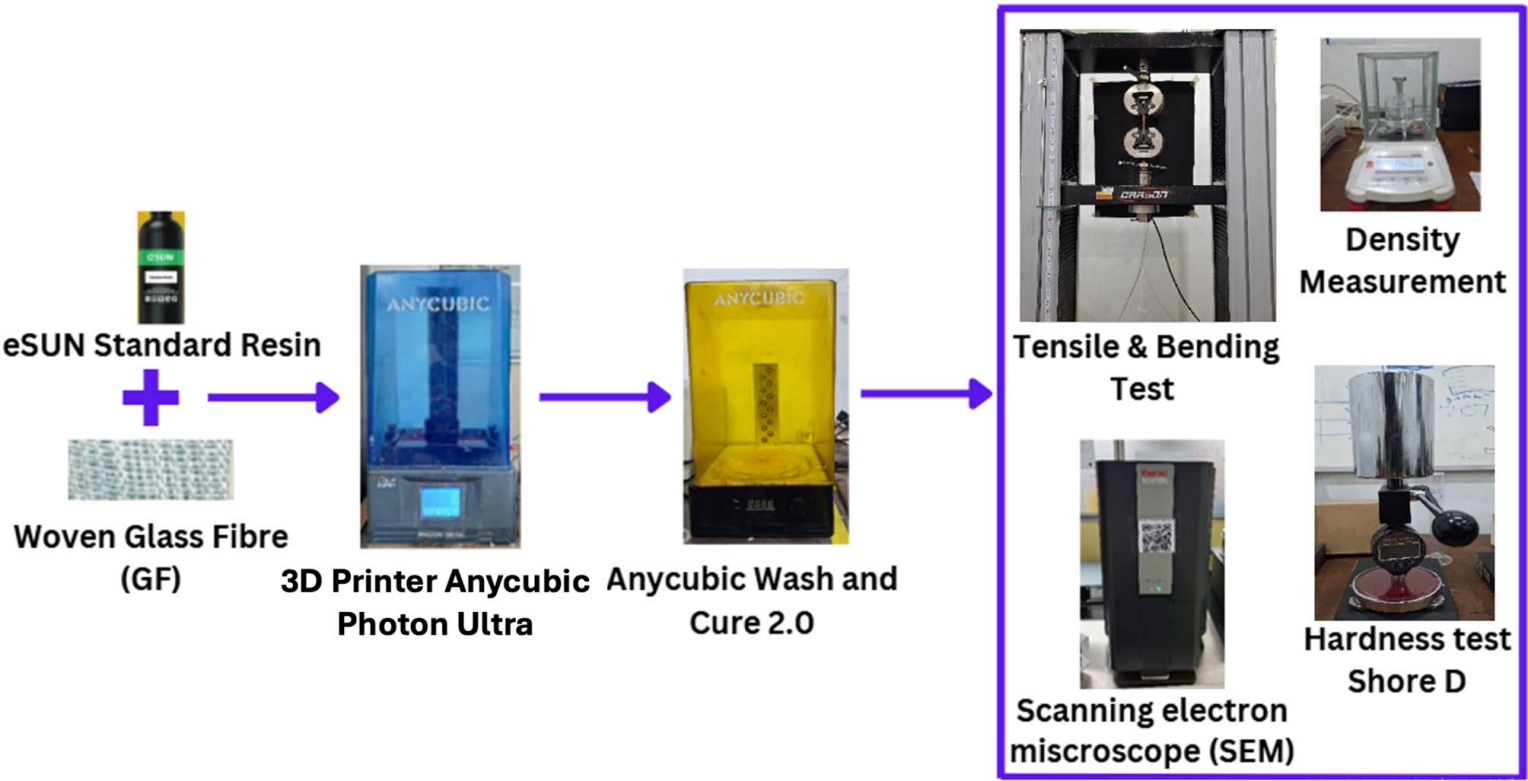
Research flow chart.

Mechanical properties evaluation was conducted using a Universal Testing Machine (UTM) Carson CRN-50, while microstructure analysis was carried out with a Scanning Electron Microscope (SEM) Thermo Scientific Phenom Pro X, following a coating treatment with a Luxor Au coating tool until a gold layer of 10 μm thickness was formed on the specimen surface. Additionally, the experiments were validated through the Digital Image Correlation (DIC) process, using a Canon 750D camera to capture the speckle pattern visualization on the specimen with high precision, allowing for accurate deformation data acquisition. The process of making glass fiber (GF) reinforced composite materials with various types of tests carried out can be seen in Fig. 1.

### 3D printing system

Autodesk Inventor software was used to design specimens with geometries that complied with mechanical test standards, such as ASTM D3039 for the tensile test, ASTM D790 for the flexure test, ASTM D792 for the density test, and ASTM D2240 for the hardness test. the effect of UV curing on the mechanical strength of resin produced using VPP technology. The files were processed using the Anycubic Photon Ultra Workshop to set the molding parameters, including layer thickness and UV light exposure time. The interaction of UV light with the resin during the curing process was identified as a crucial factor in determining the performance of the composite. The results showed that a UV wavelength of 405 nm yielded the highest tensile strength and the best elastic modulus. For post-curing, the use of this wavelength significantly influenced the achievement of optimal mechanical properties. Furthermore, increasing the temperature during post-curing not only accelerated the curing process but also enhanced the material’s overall strength and stiffness^35^. A layer thickness of 0.5 mm was found to be effective, as it allowed for more uniform UV penetration and stronger interlayer bonding. Conversely, thicker layers tended to result in uneven curing and decreased mechanical performance. An optimal UV exposure duration of 20 min was employed in the study. Insufficient exposure time could lead to incomplete curing, while prolonged exposure risked over-curing, which might cause internal stress or reduce the flexibility of the material^35^. The efficiency of this process can be affected by the characteristics of the glass fibers and their arrangement in the matrix^36^.

The ability of UV light to penetrate composite materials is critical to ensure complete drying, thus directly affecting the mechanical properties of the final product. Optimizing the weave and density of glass fibers not only improves mechanical performance but also ensures an effective drying process through increased UV light penetration^37^. In this study, after the printing process was completed, the samples were washed and cured using the Anycubic Wash and Cure 2.0 machine. The washing process was carried out for 5 min to remove any uncured resin, followed by a 5-minute post-curing process to maximize molecular bonding within the resin and enhance hardness and dimensional stability. The post-curing process plays a significant role in improving adhesion between the glass fibers (GF) and the resin matrix in 3D printing using VPP technology. Good adhesion between GF and the resin is essential to ensure effective load transfer and improved mechanical strength of the composite. This post-curing step not only strengthens the resin structure itself but also potentially enhances the interfacial bonding between the GF surface and the resin, resulting in a more solid and mechanically durable composite structure.

**Fig. 2:**
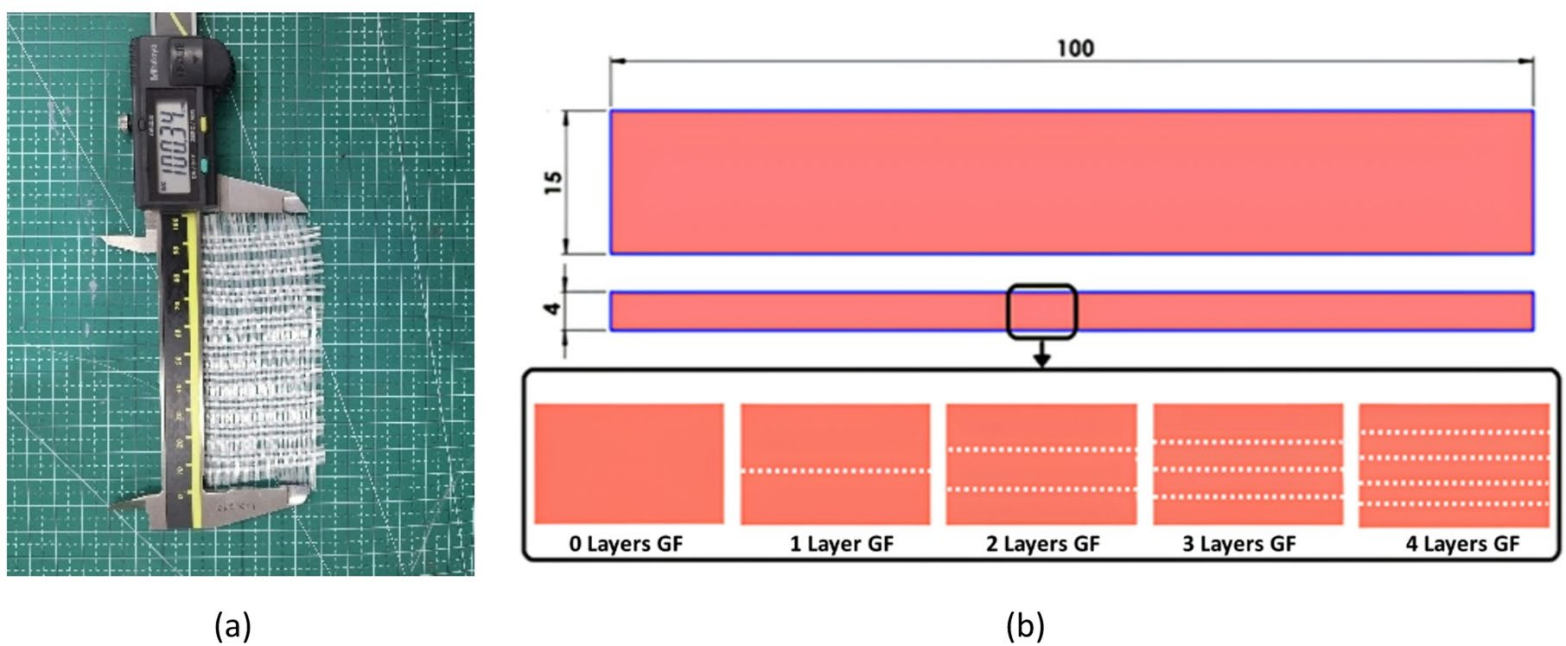
(**a**) Modified glass fiber, (**b**) Specimen dimensions and glass fiber layer variation.

Therefore, in this study, The Glass Fiber (GF) used was modified by removing one thread for every three threads in the webbing, as shown in Fig. 2a. This modification aimed to optimize the penetration of resin and UV light during the photocuring process. Improved resin infiltration ensures better wetting of the fiber surfaces, while enhanced UV light transmission promotes more uniform and deeper curing. Together, these factors contribute to stronger interfacial bonding between the fiber and the resin matrix, reducing void formation and delamination risk, ultimately enhancing the mechanical integrity and durability of the printed composite structures. The areal density of the modified GF being 177.48 g/m. The glass fiber (GF) was prepared by cutting it to the desired size and modifying its weave structure. During the molding process, the VPP printing machine was paused at designated intervals to manually laminate the GF into specific layers, in accordance with the intended layer variation, ranging from 1 layer to 4 layers. After each lamination, the machine was resumed to continue the printing process. This procedure was repeated until the desired number of GF layers was achieved within the resin matrix.

The basic standard resin composite was made with variations in the number of GF (Glass Fiber) laminates from 0 layers (control) to 4 layers which can be seen in Fig. 2b. When more than 4 layers of fiberglass (GF) reinforcement are added, the spacing between the layers becomes very close, which can lead to a reduction in the bonding strength between the matrix and the fibers. This occurs because excessively tight layering reduces the effectiveness of resin penetration into the fibers, resulting in less optimal cohesive and adhesive bonding. Additionally, the excessive number of layers significantly increases the specimen thickness. The increased thickness can cause errors in mechanical analysis, especially in tests sensitive to specimen dimensions, such as flexural and tensile tests. Inconsistent thickness may also lead to uneven stress distribution and increase the risk of localized deflection or premature failure.

**Fig. 3:**
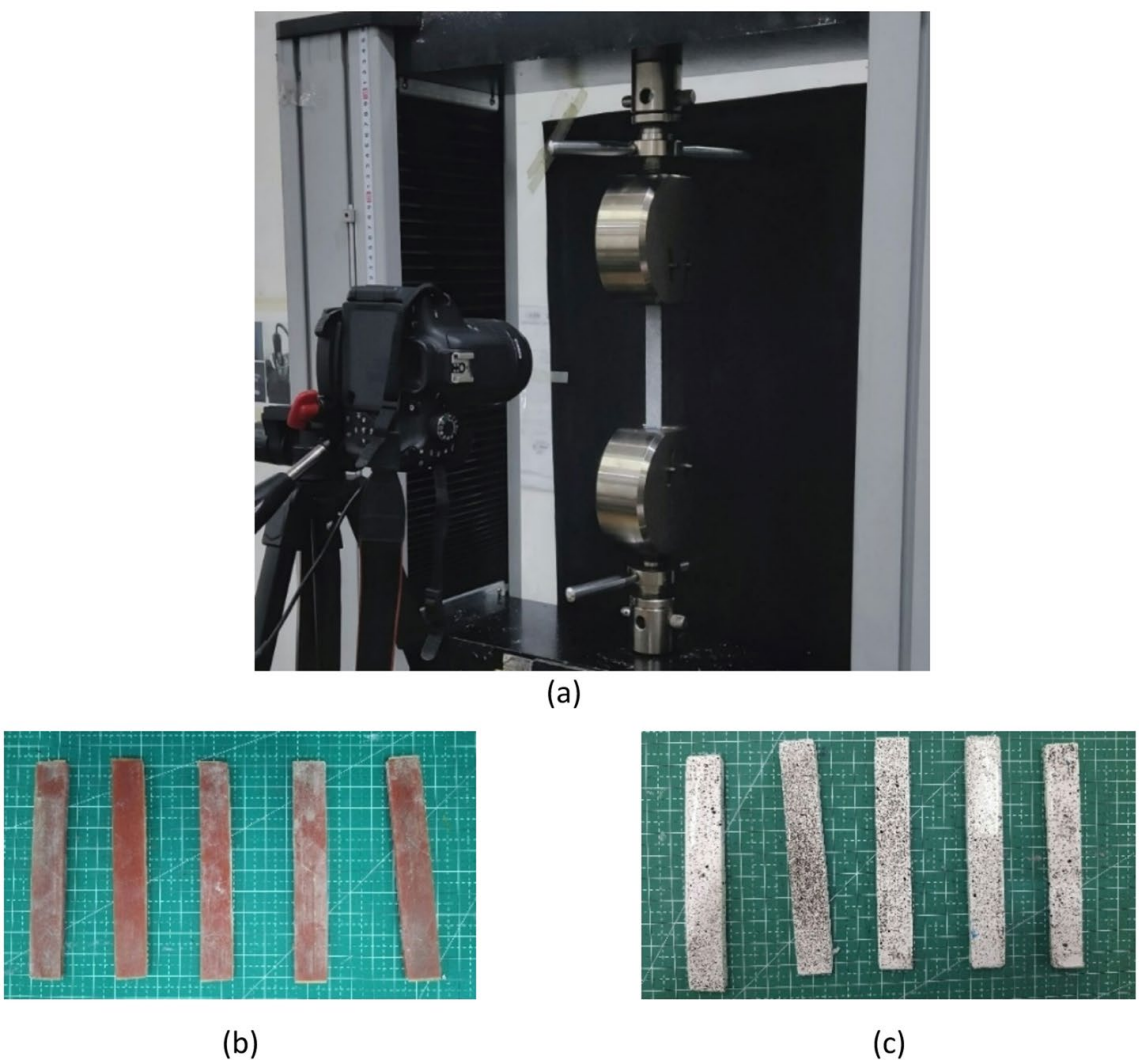
(**a**) Digital image correlation (DIC) process, (**b**) Raw samples, (**c**) Sample coloring of white and black dots for DIC.

### Testing parameters and measurement

Tests were conducted to evaluate the mechanical properties of the specimens, including tensile tests to measure the ultimate tensile strength, Flexural tests using the three-point Flexural method to determine the maximum Flexural strength, density tests to compare the density of composite materials with pure resin, and hardness tests using the Shore D tool. The tensile test in this study followed the ASTM D3039 standard. The speed used on the Universal Testing Machine, in accordance with the standard, was set at 2 mm/minute, with an initial gauge length of 60 mm and a grip length of 20 mm each. Data were collected five times. The flexural test adhered to the ASTM D790-03 standard, with a testing speed of 2 mm/min. In this test, a support span was used as a reference for placing the fulcrum, with a specimen support span length of 64 mm. A total of five specimens were tested.

The density test was conducted based on the ASTM D792 standard, where the minimum volume of the specimen used was 1 cm^3^ with unrestricted geometry and size. The specimens were weighed in air and water, and the scale automatically calculated their density. The test was performed three times for each variation, and the average results were calculated. The Shore D hardness test was conducted to determine the hardness of rigid or hard polymers, following the ASTM D2240-15 standard. According to this standard, the specimen was required to have a minimum thickness of 6 mm. In this test, hardness was measured at three points, and the average result was calculated.

### Validation testing

Validation was performed to ensure the reliability of the data obtained from the test results. Digital Image Correlation (DIC) analysis, conducted using a Canon 750D camera and MATLAB software, produced strain maps and captured specimen displacement during the tensile test. Meanwhile, Finite Element Analysis (FEA) simulation, performed using Abaqus CAE software, modeled the specimen’s behavior under tensile load. Digital Image Correlation (DIC) is a technique used to generate strain and displacement maps of a specimen during a tensile test. DIC works by comparing digital images of the specimen at two different times, so as to measure the changes that occur due to deformation. The process involves capturing images with a camera and analysis using software to process the data obtained^38–40^. DIC has the advantage of generating displacement and strain plots with high accuracy. A study shows that DIC can be used to detect and monitor surface displacements using multitemporal digital images, which enables a more in-depth analysis of specimen behavior under load^38^. In DIC analysis, the variety of specimens used, such as specimens without a glass fiber coating, provides valuable insight into the behavior of the material when tested. Previous research has shown that DIC can provide better information compared to other measurement techniques, especially in noisy environments^41^. Overall, in this study, the use of DIC in tensile testing provided reliable and accurate data, which was crucial for validating the analysis results. By utilizing this technology, researchers were able to generate better information regarding material characteristics and deformation behavior, which in turn improved the understanding of material mechanics. This demonstrated that DIC was not only effective in displacement measurements but also in identifying strain patterns that occurred in specimens during testing. Specimen preparation for the DIC process as well as video capture can be seen in Fig. 3.

Finite Element Analysis (FEA) simulations carried out using software such as Abaqus CAE played an important role in modeling the behavior of the specimen under tensile load. FEA enables in-depth numerical analysis and can be used to validate the results obtained from DIC. By combining the results from DIC and FEA, researchers can gain a more comprehensive understanding of the material behavior and deformation mechanisms that occur^42–44^. The tensile test simulations were validated using Abaqus software. Materials were defined based on their mechanical properties, such as density, elasticity, plasticity, and fracture characteristics, and were then simulated. The material variations used in the simulation were adjusted according to the number of glass fiber layers in the VPP composite, namely 0 layers, 1 layer, 2 layers, 3 layers, and 4 layers. The experimental data were input into Abaqus to define the material properties using the hyperelastic material feature. The Mooney-Rivlin, Polynomial, Neo-Hookean, Yeoh, and Ogden constitutive models were selected to align with the test results. Data plots for the uniaxial tensile test were then obtained^45^. Simulation results, including stress distribution visualizations, numerical data, and graphs, were analyzed. A comparison between the experimental and simulation results was conducted, and if the error difference did not exceed 5%, the data was considered valid and used for analysis.

## Results and discussion

### Manufacturing results

**Fig. 4:**
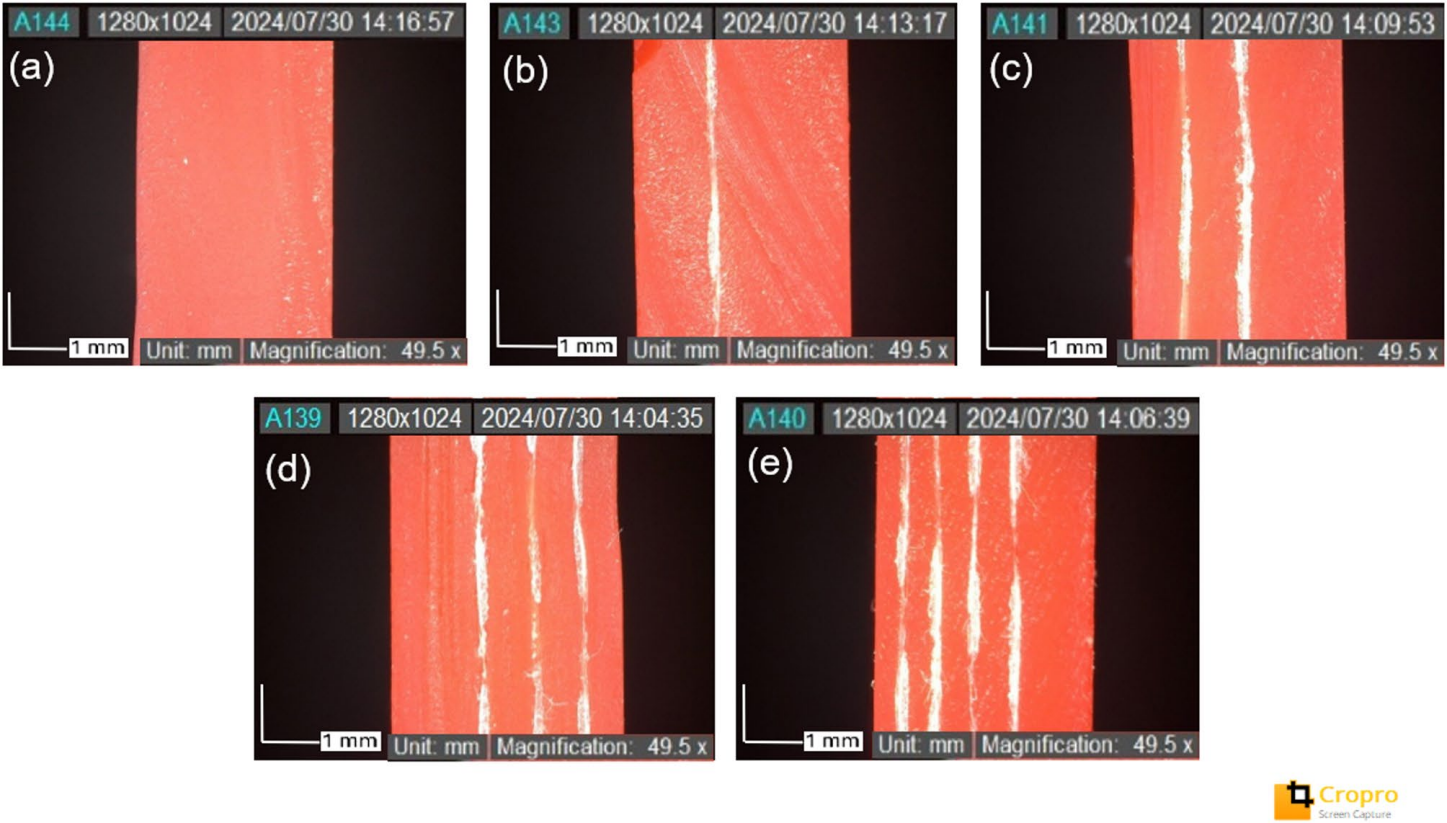
GF-VPP laminate manufacturing results: (**a**) 0 layer, (**b**) 1 layer, (**c**) 2 layers, (**d**) 3 layers, (**e**) 4 layers.

The results of manufacturing GF-reinforced VPP standard resin composite specimens with variations of 0 layers, 1 layer, 2 layers, 3 layers, and 4 layers can be seen in Fig. 4. where it can be seen that the GF layer placement is not symmetrical in the resin matrix, the asymmetry of the distribution of glass fiber (GF) layers in the composite structure resulting from the 3D stereolithography process with GF reinforcement, such as the asymmetrical thickness of the matrix between layers, occurs due to the limitations of the manufacturing process, this occurs when the laying of GF in the resin matrix is done manually. The asymmetrical GF layers in the resin matrix indicate limitations in maintaining geometric consistency during the fabrication process, especially at the micro scale where the photopolymer resin flows before it is fully sintered and cured. Such asymmetry could potentially lead to uneven internal stress gradients when the composite is subjected to flexural loading. Non-uniform stress distribution can result in localized stress concentrations, lowering the maximum load capacity of the material, as well as increasing the likelihood of inter-layer delamination. This is evidenced by the decrease in bending stress along with the addition of layers as shown in Fig. 11. Further research can be developed on more precise reinforcement placement techniques, such as the integration of robotic or automation methods in GF layering, this aims to minimize the influence of operator subjectivity and improve the internal symmetry of the reinforcement layer in the resin matrix.

**Fig. 5:**
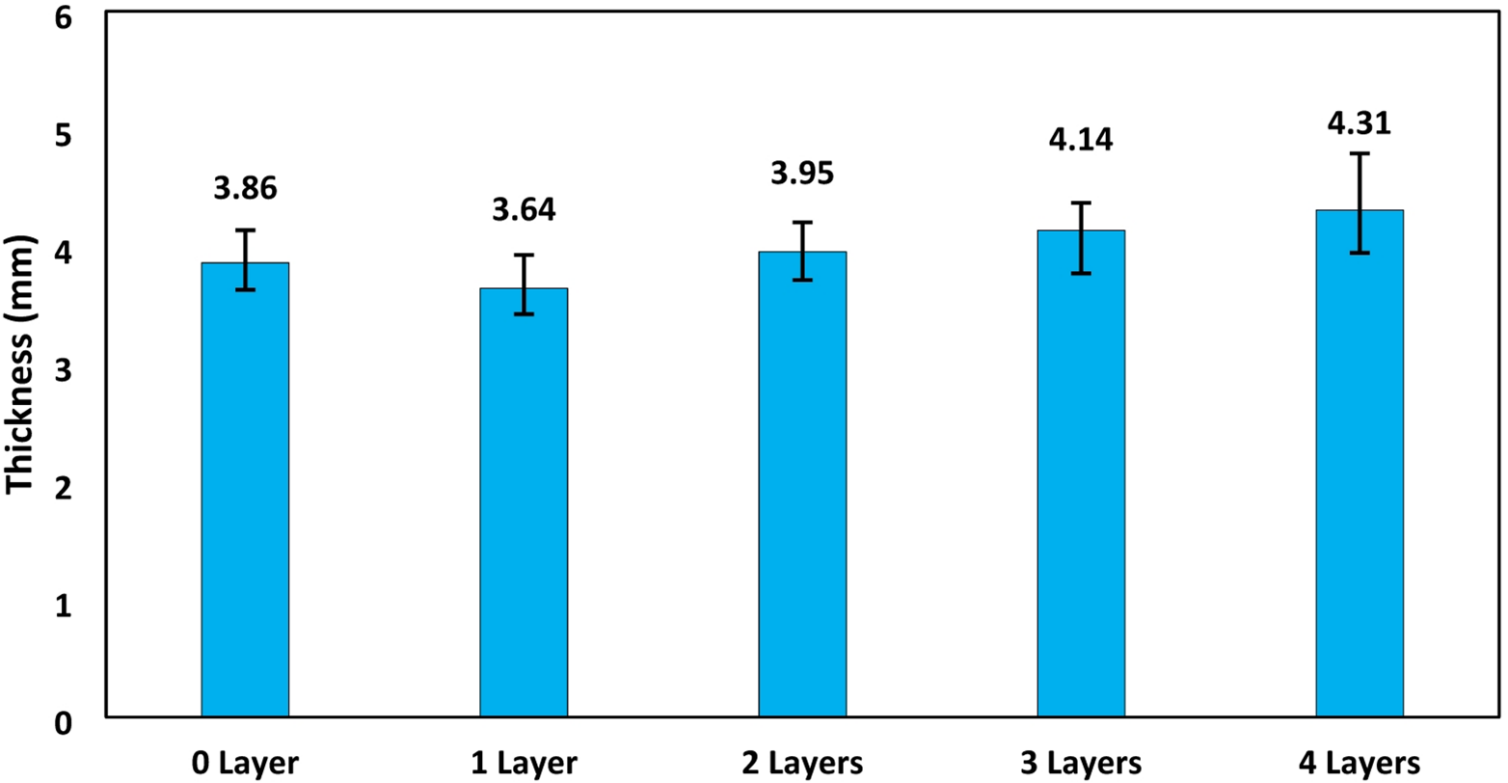
Specimen thickness at various GF layers.

The thickness of the specimens in the various variations performed had differences, as shown in Fig. 5. The thickness of the specimens tended to increase as the number of glass fiber layers increased. In specimens that used glass fiber, the resin was absorbed by the glass fiber. As a result, the specimen with 1 layer of glass fiber was thinner than the specimen with 0 layers of glass fiber, then with additional layers of glass fiber, the thickness tends to increase when compared to specimens without additional glass fiber reinforcement. The thickness value of the specimen with 0 glass fiber layer is 3.860 mm, 1 glass fiber layer = 3.643 mm, 2 glass fiber layers = 3.953 mm, 3 glass fiber layers = 4.136 mm, 4 glass fiber layers = 4.309 mm.

**Table 1:** GF fraction difference calculation.

GF layer	Real mass fraction		Theoretical mass fraction		GF fraction difference [Real-Theoretical]
	Resin	GF	Resin	GF	
0 layer	100.0%	0.0%	100.0%	0.0%	0.00%
1 layer	92.6%	7.4%	92.2%	7.8%	0.40%
2 layers	86.7%	13.3%	86.0%	14.0%	0.78%
3 layers	81.4%	18.6%	80.2%	19.8%	1.16%
4 layers	76.7%	23.3%	75.1%	24.9%	1.52%

The specimen thickness changes along with the addition of GF reinforcement to the matrix made by the VPP 3D printing method. The addition of GF will certainly increase the thickness of the composite, to assess whether the resin impregnates the GF reinforcement structure with a consistent phenomenon in each variation of the GF reinforcement layer, it can be assessed by calculating the difference between the real and theoretical fiber fractions. In this study, the gap between real and theoretical fiber fraction tends to increase as the number of layers increases, as shown in Table 1. Where the Fiber Fraction Difference rises from 0.4% in GF single-layer reinforced composites, and continues to grow to 1.52% in GF 4-layer reinforced composites. The main cause is because some of the resin can be absorbed in the microscopic structure of GF, the more layers of GF, the more resin is absorbed, which is shown in the increase in the real and theoretical Fiber Fraction Difference which is getting bigger as the GF reinforcement is added. This phenomenon causes the overall recorded resin mass to be greater than the theoretical resin mass, and the real fiber fraction to be lower than the theoretical fiber fraction. Although some of the resin can fill the micro-cracks of the GF structure, the resin absorption occurs unevenly throughout the GF microscopic structure, the resin liquid cannot wet and absorb to reach the bottom of the GF microscopic structure. When the fiberglass layer is placed on the surface of the VPP material, the resin is more easily absorbed and fills the pores of the layer at the top of the GF structure, while the lower layer is less impregnated.

### Tensile test results

In the variation without glass fiber or with 0 layers, the composite material tended to be elastic, resulting in a relatively large strain. However, the material was less capable of withstanding high stress. Meanwhile, in variations with more glass fiber layers, the material’s ability to withstand loads generally increased as the number of layers increased, along with the strain value. This phenomenon also occurred in previous studies, where the addition of FG layers increased the tensile strength of the material^46^. This occurred because the loading in the tensile test took place in the axial direction, allowing all layers of glass fiber to contribute optimally in resisting the load. Consequently, the tensile strength of the specimen increased as the number of glass fiber layers increased.

**Fig. 6:**
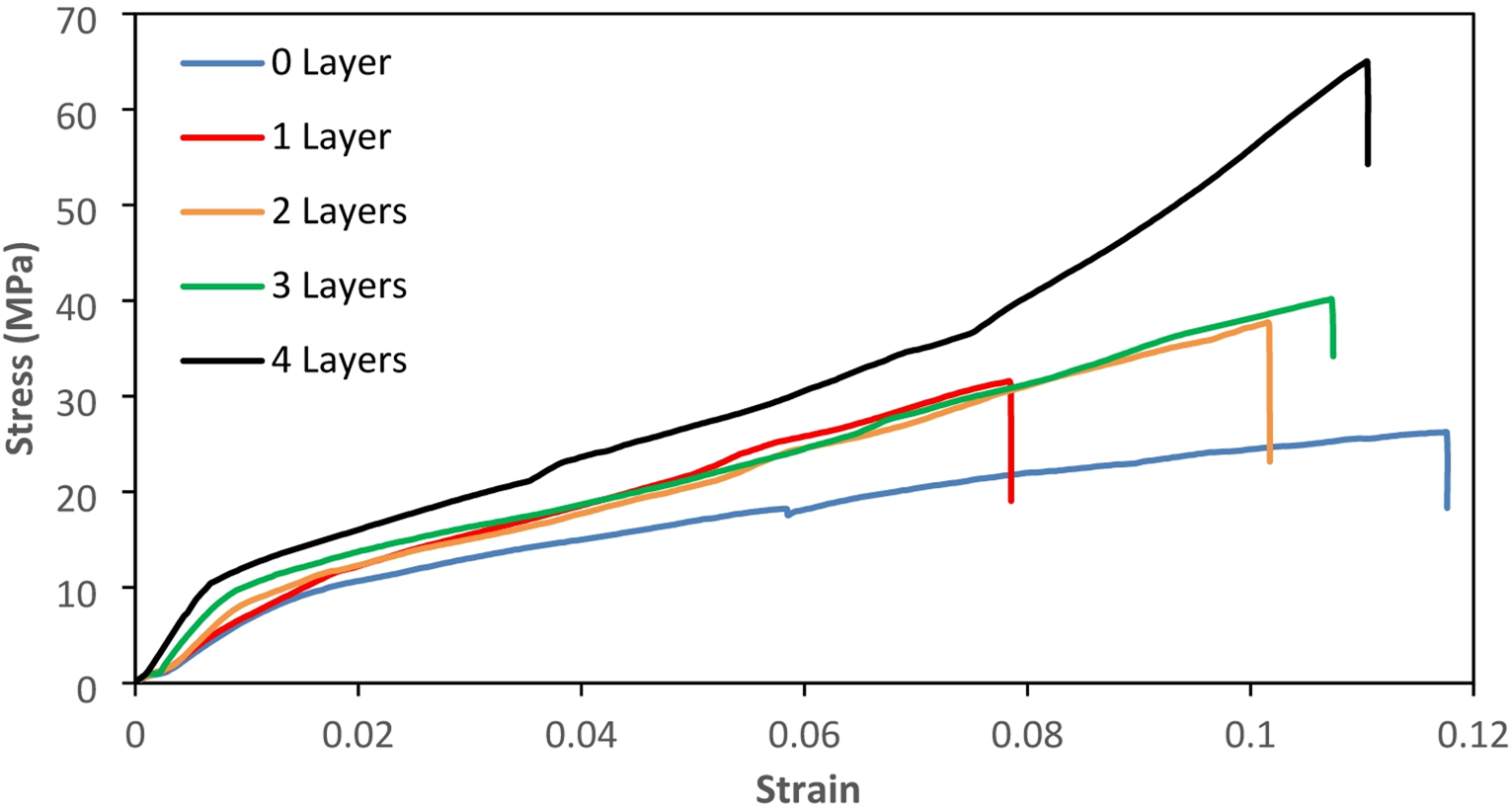
Stress-strain curve of the tensile test results.

Additionally, the fibers in the material were arranged parallel to the direction of the tensile force (unidirectional orientation), which is the most effective alignment for resisting axial loads, allowing maximum stress transfer from the matrix to the fibers. The increasing number of layers not only increased the fiber volume fraction but also reduced stress concentration within the matrix, thereby improving overall mechanical integrity. However, as the fiber content increased, the failure behavior of the composite also shifted—from matrix-dominated failure (in 0 and 1 layer specimens) to fiber pull-out, interfacial debonding, and eventual fiber breakage in the specimens with 3 and 4 layers. These failure modes are typical in fiber-reinforced composites and indicate more efficient stress distribution and energy absorption. The stress-strain curves for the variations of 0 layer, 1 layer, 2 layers, 3 layers, and 4 layers can be seen in Fig. 6.

**Fig. 7:**
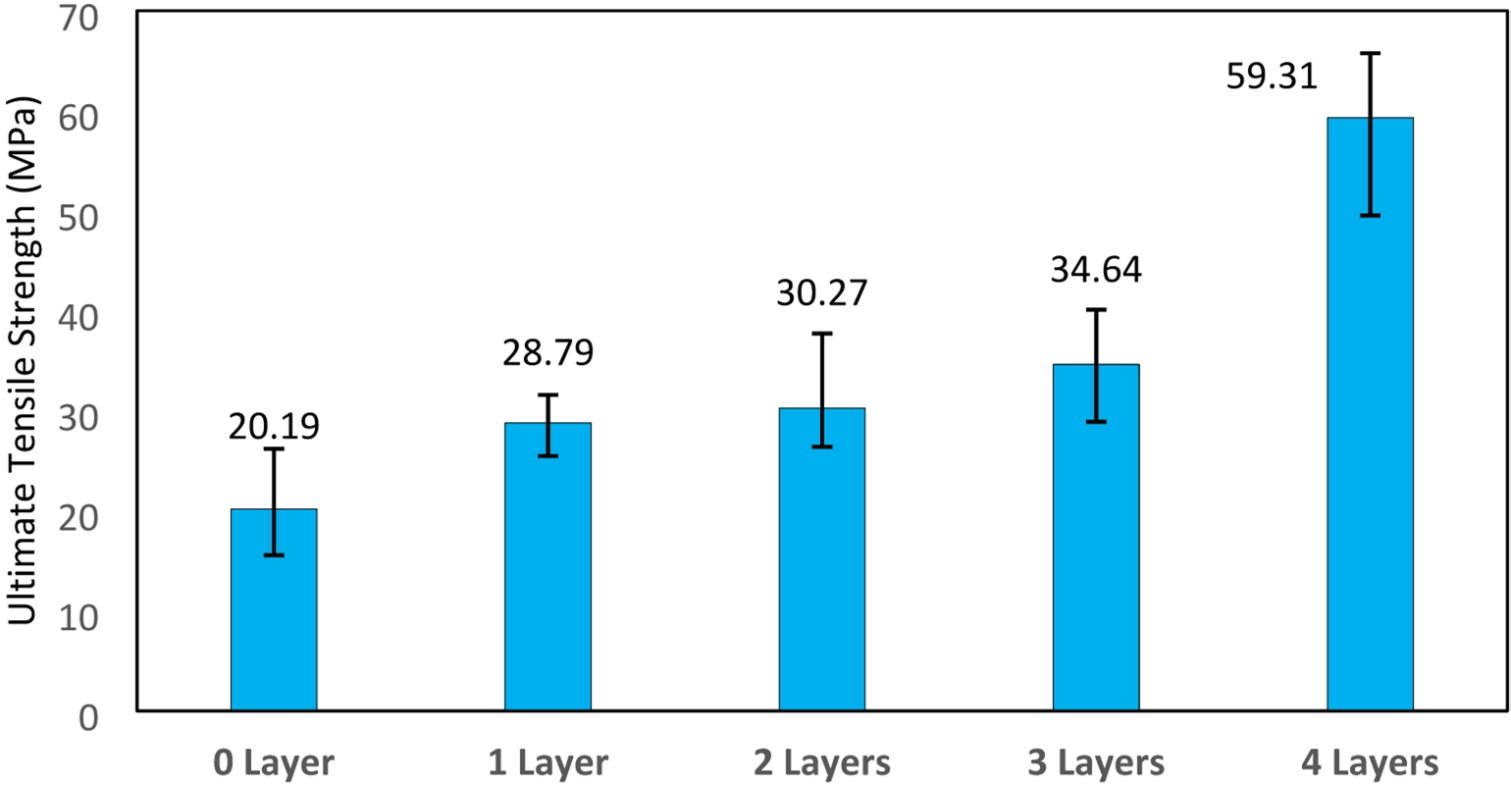
Ultimate tensile strength results.

The ultimate tensile strength (UTS) in each variation increased as the number of fiberglass layers in the composite increased. In the variation without layers, the average UTS was 20.1 MPa. With one layer, the average UTS increased to 28.7 MPa. For the variation with two layers, the average UTS reached 30.2 MPa. Furthermore, with three layers, the average UTS was 34.6 MPa, and with four layers, it reached 59.3 MPa, this result can be seen in Fig. 7. This trend further confirms that the reinforcement effect becomes more prominent as the fiber content increases, especially when fibers are well aligned with the loading axis. The increase in tensile strength for composites reinforced with fiberglass (GF) layers from 1 to 3 is relatively small compared to the improvement observed with 4 layers. This is because a greater number of fiberglass layers provides a stronger correlation to the increase in the material’s tensile strength. As the number of fibers increases, the bonded surface area between the matrix and the reinforcement also expands, enhancing the effective load transfer from the matrix to the fibers. This allows for a more uniform stress distribution and a higher overall tensile capacity of the composite. Additionally, the increased fiber content strengthens the internal network of the composite, reducing the likelihood of premature failure due to cracking or delamination. Therefore, using 4 layers of fiberglass reinforcement results in a significantly greater improvement in tensile strength compared to fewer layers, making the number of layers a crucial parameter in the design and optimization of fiberglass-based composite materials.

The increase in tensile strength was nearly threefold, as specimens without reinforcement had an average UTS of 20.1 MPa, while specimens with four layers of fiberglass had an average UTS of 59.3 MPa. This result is similar to the research of composites with photosensitive resin matrix 3D printed VPP with reinforcement woven carbon fiber which can increase the tensile strength up to 3 times greater^32^. Such improvements are often attributed not only to the mechanical properties of the fibers themselves but also to the reduction of premature matrix cracking and delamination due to better fiber bridging and stress redistribution.

**Fig. 8:**
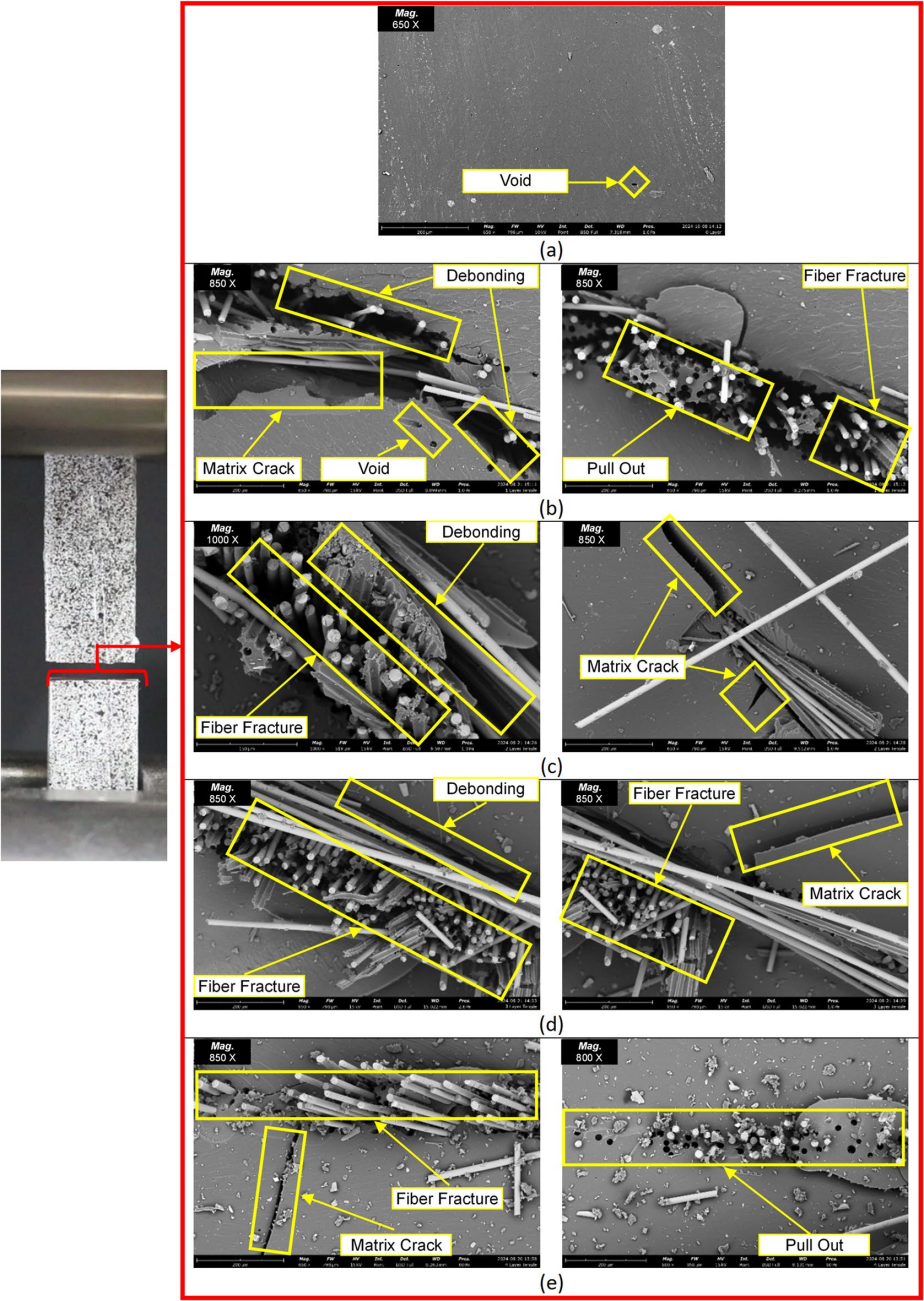
SEM test results on fiberglass reinforcement variations: (**a**) 0 layer, (**b**) 1 layer, (**c**) 2 layers, (**d**) 3 layers, (**e**) 4 layers.

SEM testing was conducted on the tensile test specimens, particularly in the failure area, to determine the type of failure that occurred. In the fracture analysis of the tensile test fracture results with a 0-layer glass fiber variation, it is clear that the type of damage is the fracture of the resin matrix, with a relatively straight fracture pattern in the failure area. Meanwhile, for specimens with glass fiber layer variations from 0 to 4, the types of failure experienced by the tensile test specimens exhibit similar damage patterns. The failures occurring in specimens with glass fiber layer variations from 0 to 4 include matrix cracks, fiber fractures, pull-out, and debonding. In previous studies, it was also reported that laminated materials are dominated by damage mechanisms such as delamination, buckling, and fiber fracture^47–49^. This failure pattern can be seen in Fig. 8.

In the failure analysis of composite materials with VPP resin matrix reinforced by glass fibers, fracture observations revealed failures along the transverse GF fiber direction. This indicates an early sign of significant structural weak points within the composite material. Transverse fibers, which are oriented perpendicular to the main tensile load direction, act as stress concentrators when the material is subjected to tensile loading. Unlike longitudinal fibers, which primarily bear the load, transverse fibers contribute less to axial strength (i.e., force parallel to the longitudinal axis). As a result, the arrangement of transverse GF fibers can lead to an imbalance in stress distribution within the composite structure, causing stress accumulation around the transverse fibers and potentially initiating early cracks, which may lead to premature failure.

This phenomenon is also closely associated with delamination mechanisms and interfacial debonding between fibers and the matrix. Delamination typically arises from the inability of composite layers to withstand interfacial shear stress, while debonding reflects weak adhesion between fibers and the matrix—particularly in areas containing micro-defects or irregularities. Misaligned transverse fibers not only exacerbate this condition but can further increase the risk of damage, especially if they are unevenly distributed or already locally degraded.

In multilayer glass fiber-reinforced composite structures with a resin matrix produced through VPP, the failure mechanisms are strongly influenced by fiber orientation (longitudinal and transverse), the fiber weave pattern, and the interfacial bonding quality between the fibers and the matrix. In woven structures, fiber intersection points are potential sites for stress concentration and resin impregnation defects, which accelerate damage initiation. When the composite structure is subjected to axial loads, such as in tensile testing, failure typically begins with the formation of microcracks in the resin matrix (matrix cracking) due to stresses exceeding the matrix’s elastic limit. These cracks tend to propagate between fiber bundles oriented transversely, as transverse fibers are less effective in bearing axial loads compared to longitudinal fibers. Cracks in the matrix then lead to stress concentrations at the fiber–matrix interface, which may result in debonding or separation of the bond between the fibers and the matrix. If the bonding is weak or manufacturing defects are present, fibers—especially those oriented transversely—may be pulled out of the matrix without breaking (fiber pull-out). As the load increases, longitudinally oriented fibers—the main load-bearing elements—may eventually fracture when their tensile strength is exceeded. Fiber fracture is the most critical form of failure, as it marks the end of the composite’s load-bearing capability. This entire process illustrates that failure in composites is progressive and interconnected, starting from weaknesses in the matrix and the interface, and culminating in total structural failure due to fiber rupture^31,50^.

**Fig. 9:**
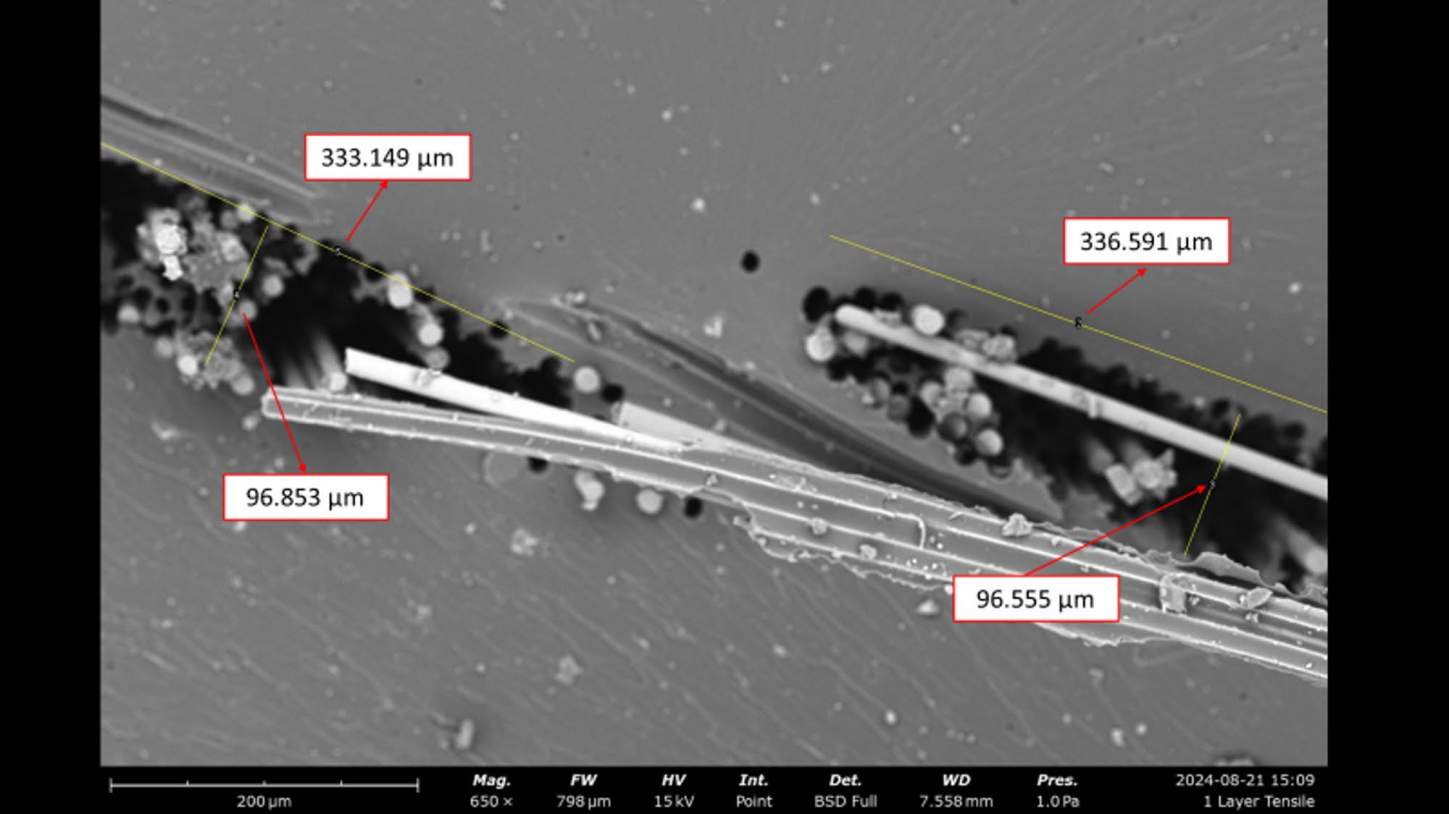
The analysis of crack length and width in SEM testing.

The analysis of crack length and width in SEM testing is essential for understanding the material’s failure mechanism at the microscopic level. Crack dimensions provide important information on how far the crack has propagated due to the applied load, as well as how the microstructure—such as the matrix and fibers—interacts during failure. A significant crack length may indicate rapid crack initiation and the potential for delamination, while crack width can reflect the degree of local deformation. The results of this analysis can be observed in Fig. 9.

Figure 9 is a Scanning Electron Microscope (SEM) image at 650x magnification of a specimen with a 1-layer glass fiber reinforcement variation, showing the surface of the material after undergoing tensile testing. Crack length and width were analyzed using ImageJ software. Based on the scale bar of 200 μm, a visual analysis was performed on the main crack, which appears clearly extending diagonally across the center of the image. The measured crack lengths were 336.591 μm and 333.149 μm, while the crack widths were 96.853 μm and 96.555 μm. However, the SEM’s visual area cannot capture the entire crack, so it can be concluded that the actual crack length is significantly greater than the measured values. The observed crack appears as an elongated gap, indicating the possibility of interfacial cracking or fibrous fracture, suggesting a failure mechanism such as delamination or fiber pull-out. The substantial crack length indicates that, after initial crack initiation, rapid crack propagation occurred due to the applied tensile force—especially in the direction where the fibers are aligned parallel to the applied load during testing.

### Flexural test results

**Fig. 10:**
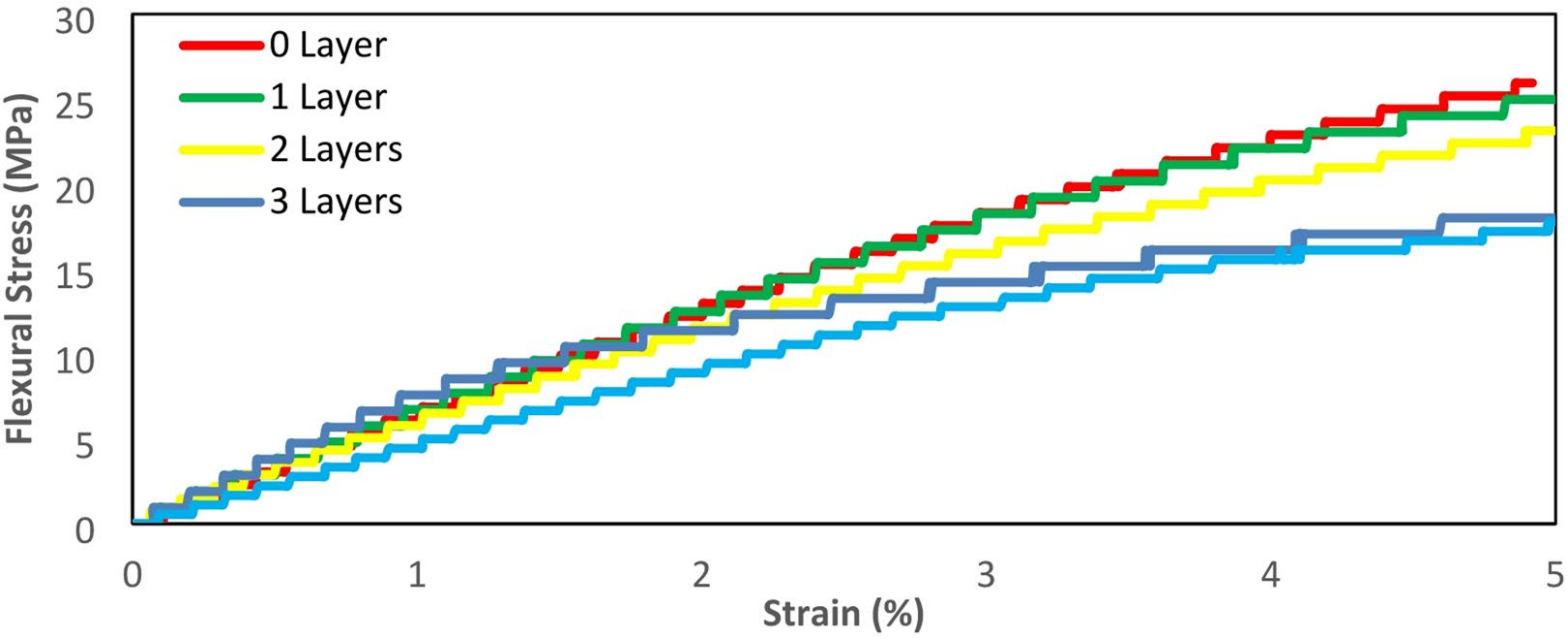
Flexural stress-strain curve.

In this study, it was found that as the number of glass fiber layers increased, the structure’s strength to withstand Flexural loads decreased, can be seen in Fig. 10. Flexural stress-strain curve. This phenomenon occurred because the adhesion factor between the glass fiber and the eSUN Standard resin matrix was not good, as evidenced by the numerous failures due to debonding observed in the SEM analysis of the tensile test fracture results. Furthermore, the orientation or direction of the glass fibers relative to the loading axis plays a crucial role in the load-bearing capacity of the composite. In this study, the fiber layers were oriented predominantly in the same direction, which means that with more layers added, the risk of interlayer weaknesses increased due to poor adhesion, especially under flexural loading where stresses vary through the thickness. As a result, when the number of fiber layers increased, the weak adhesion strength between the fiber and the matrix led to a reduction in flexural strength. Previous studies have also reported that poor adhesion between fibers and the resin matrix can significantly affect the flexural strength of composite materials. This is because weak interfacial bonding reduces the load transfer strength between the fiber and the matrix, leading to delamination before more severe damage occurs^51–55^. In Flexure loading, the force was applied transversely to the length of the specimen, causing tensile and compressive stresses to occur on different sides. Due to the layered structure and the orientation of the fibers, the increase in layers intensified the internal stress gradients between layers, exacerbating delamination and interfacial failure. Regarding adhesion strength, when it decreased, some layers did not contribute optimally in resisting the bending stresses. Unlike the tensile test, where the loading was axial, all fiber layers could contribute effectively to withstand the load.

**Fig. 11:**
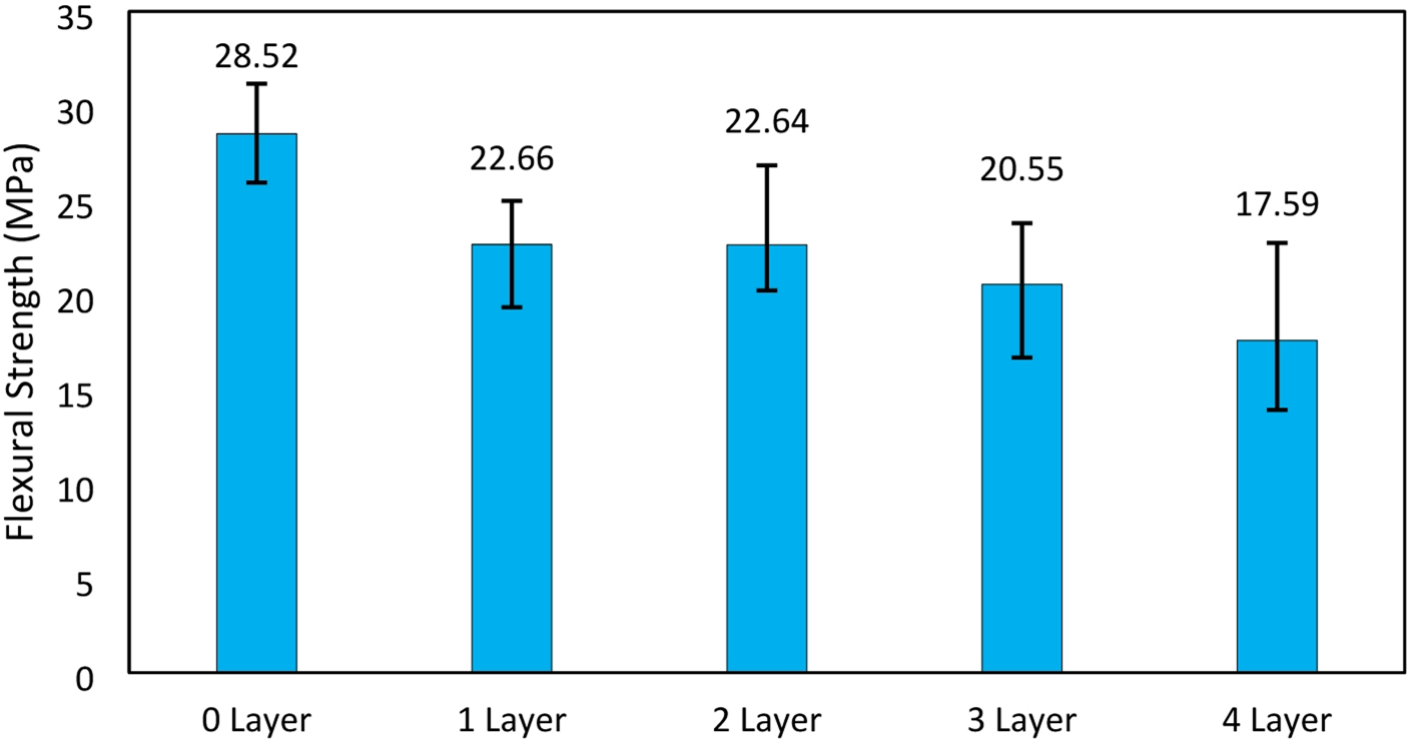
Maximum flexural strength.

The maximum flexural strength of each variation tended to decrease as the number of glass fiber layers increased, as described in the previous paragraph. This differs from the results of the flexural test in the study by Song et al., where the flexural strength increased in composite materials reinforced with fiberglass. However, the addition of a silane coupling agent may have contributed to the increase in bending strength due to the enhanced cohesive bonding within the resin and the adhesive bonding between the resin and fiberglass^30^. Based on the test results, the 0 layer variation had the highest average maximum flexural strength of 28.52 MPa, followed by the 1 layer variation with 22.66 MPa, the 2 layer variation with 22.64 MPa, the 3 layer variation with 20.55 MPa, and the 4 layers variation with 17.58 MPa. The maximum flexural strength results can be seen in Fig. 11.

**Fig. 12:**
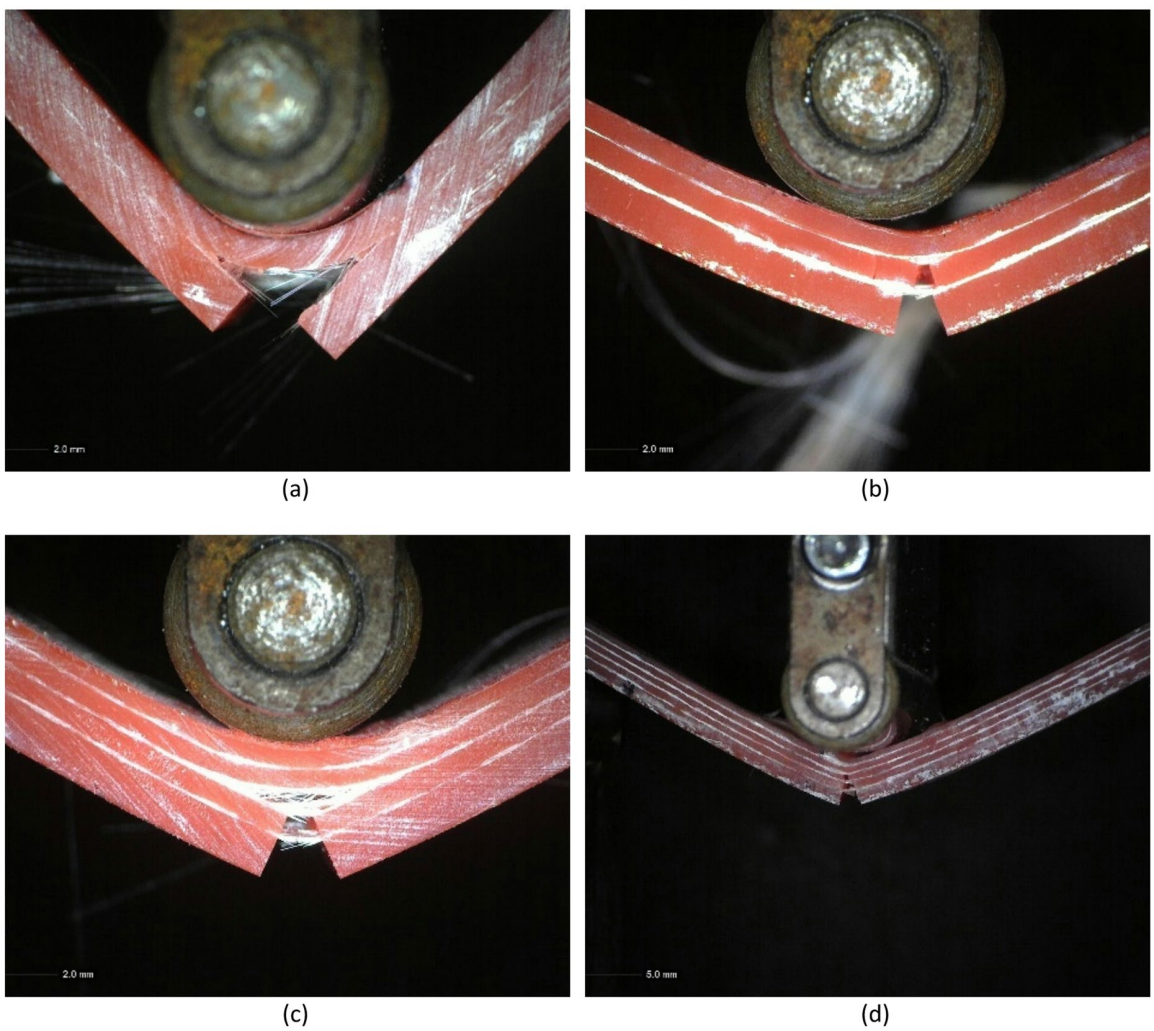
Fracture results of the flexural test: (**a**) 1 layer, (**b**) 2 layers, (**c**) 3 layers, (**d**) 4 layers.

The results of the fault analysis in the curvature test showed that the specimens tended to delaminate in the middle fiber layer. This occurred when the composite was subjected to flexural loading, where the outer layers experienced different tensile and compressive stresses, while the middle layer experienced maximum shear stress. This triggered high interlayer stresses, especially near the center of the laminate. During the flexural test, the top surface of the specimen experienced a compressive force, while the bottom surface experienced a tensile force, ultimately causing the material to break due to the tensile stress, as shown in Fig. 12.

This failure mode, delamination, is strongly influenced by the fiber orientation and the number of layers, where more layers lead to increased interfaces susceptible to shear stress-induced separation. Delamination is a typical failure mode in layered composites, greatly impacting their mechanical performance and potentially causing structural collapse. Under bending loads, varying stress distributions occur across the layers, with the central layer experiencing the highest shear stress, which may initiate delamination^56–58^. Delamination occurred due to the stress distribution in the middle part, particularly in the laminated area, which experienced shear stress in opposite directions. During the curvature test, the upper side of the specimen was subjected to compressive stress, while the lower side experienced tensile stress. As a result, maximum shear stress developed in the middle layer of the composite. This shear stress caused the separation of layers if the adhesion strength of the composite was not sufficient. Therefore, both the poor fiber-matrix adhesion and the increased number of fiber layers, combined with the directional loading and fiber alignment, contributed to the observed delamination and reduction in flexural strength. The type of failure observed—debonding and delamination—directly correlates with these structural and material parameters, emphasizing the importance of optimizing fiber orientation and improving interfacial bonding in multilayer composites.

The study by Song et al., evaluated the enhancement of mechanical properties in VPP-based photosensitive resin composites reinforced with glass fibers. The test results showed that the addition of glass fibers increased tensile strength by up to 50% and flexural strength by up to 143%, especially when the fibers were treated with silane. Additionally, the fiber coating method with a three-dimensional orthogonal structure improved tensile strength by up to 110% and flexural strength by up to 147%, compared to the continuous long fiber coating method. These findings confirm that surface treatment and fiber orientation play a crucial role in enhancing the mechanical performance of VPP composites. Therefore, further studies are needed to investigate the tensile and flexural properties of VPP-based photosensitive resin composites reinforced with glass fibers without the addition of silane as a coupling agent^30^.

**Fig. 13:**
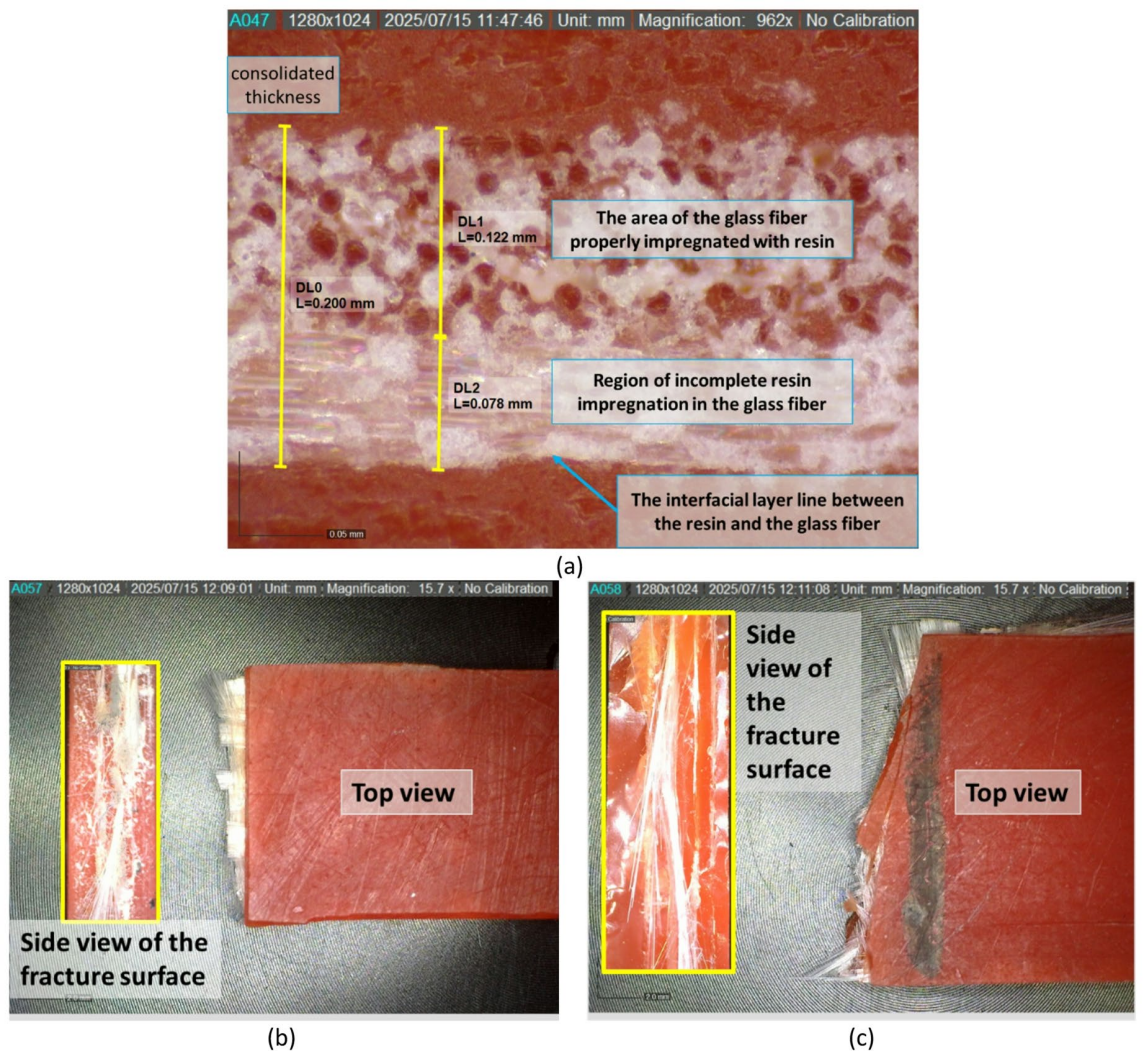
Microscopy observations on specimens: (**a**) Cross-section, (**b**) Fracture surface after tensile test, (**c**) Fracture surface after flexural test.

In the flexural test, a larger number of GF layers in the composite causes a decrease in strength, which is due to weak interfacial bonds, especially on the lower surface of the GF layer. The weakness of the resin-GF interfacial bond is evidenced by microscopic observation of the cross-section of the bonding area between the resin matrix and glass fiber reinforcement. The phenomenon of impregnation and the formation of resin adhesive bonds into the GF structure can be seen in Fig. 13, where at the bottom there is a strong boundary line between resin and GF, this is due to the limitations of the 3D VPP composite manufacturing process, namely in the process of laying the GF reinforcement layer, where the resin matrix is printed first then the 3D VPP machine is stopped to lay the GF reinforcement layer on the surface of the 3D VPP printed material. This makes the resin that has been printed only stick to the bottom surface of the GF, this process makes the resin not well absorbed in the glass fiber structure, especially in the lower surface area of the GF structure.

This phenomenon is clearly visible with the formation of a strong boundary line that is the interface layer line of resin and GF, where the thickness of the GF reinforcement area that is not well impregnated with resin reaches 0.078 mm. This area is the microscopic space of the fiber that is not filled with resin so that air can be trapped in the microscopic space. These areas make the composite structure weak because there is no adhesion and micromechanical interlocking between the resin matrix and the GF reinforcement structure. Thus, the more layers of GF reinforcement that are added, the larger the area that is not well impregnated with resin, ultimately making the flexural strength will decrease due to the weak bonding structure between the face of the resin matrix and GF reinforcement. A different effect is shown on the top of the GF reinforcement in the composite structure. After the VPP 3D printing machine was re-operated, the resin was melted and wetted the surface of the reinforcement that had been placed on the surface of the VPP material that was printed first. In Fig. 13a, the top of the GF reinforcement structure appears to be well impregnated with resin, the resin wets and permeates into the GF reinforcement structure perfectly and reaches a thickness of 0.122 mm. However, it was found that the resin could not reach and permeate the bottom of the GF structure, which eventually became the weakest area of the GF layer-reinforced VPP 3D printing composite structure.

Based on the comparison of the observation results of the specimen’s fracture surface after the tensile test and flexural test, there is a significant difference between the fracture patterns due to the tensile test and flexural test. In Fig. 13b, the fracture surface due to the tensile test shows random and asymmetrical characteristics. The glass fiber is pulled out in an irregular direction, indicating a dominant fiber pull-out phenomenon. This indicates that when the specimen is axially pulled, the tensile force causes the fibers to pull out of the matrix before complete failure. The irregularity of the fracture pattern indicates that cracks occur progressively at various weak points due to the weak interfacial bond between the resin matrix and the glass fiber. By contrast, the results of the specimen fracture after the bending test seen in Fig. 13c, show a straighter and more symmetrical pattern. This fracture follows the direction of the force on the specimen during the flexure test, where the force acting on the specimen is a combination of tensile stress on the lower side and compressive stress on the upper side of the specimen. The fracture pattern extends parallel to the direction of the load, indicating delamination between composite layers and rupture of the matrix due to compressive forces right at the point of action. The dominant failure occurs due to interlayer sliding or cracks that propagate stably in the tensile zone. This difference in pattern shows that the failure mechanism in composites is strongly influenced by the type of load applied, where tensile tests tend to produce random damage due to fiber and matrix separation, while flexural tests produce more directional and symmetrical cracks due to the complex interaction between tensile, compressive, and delamination.

### Hardness test results

**Fig. 14:**
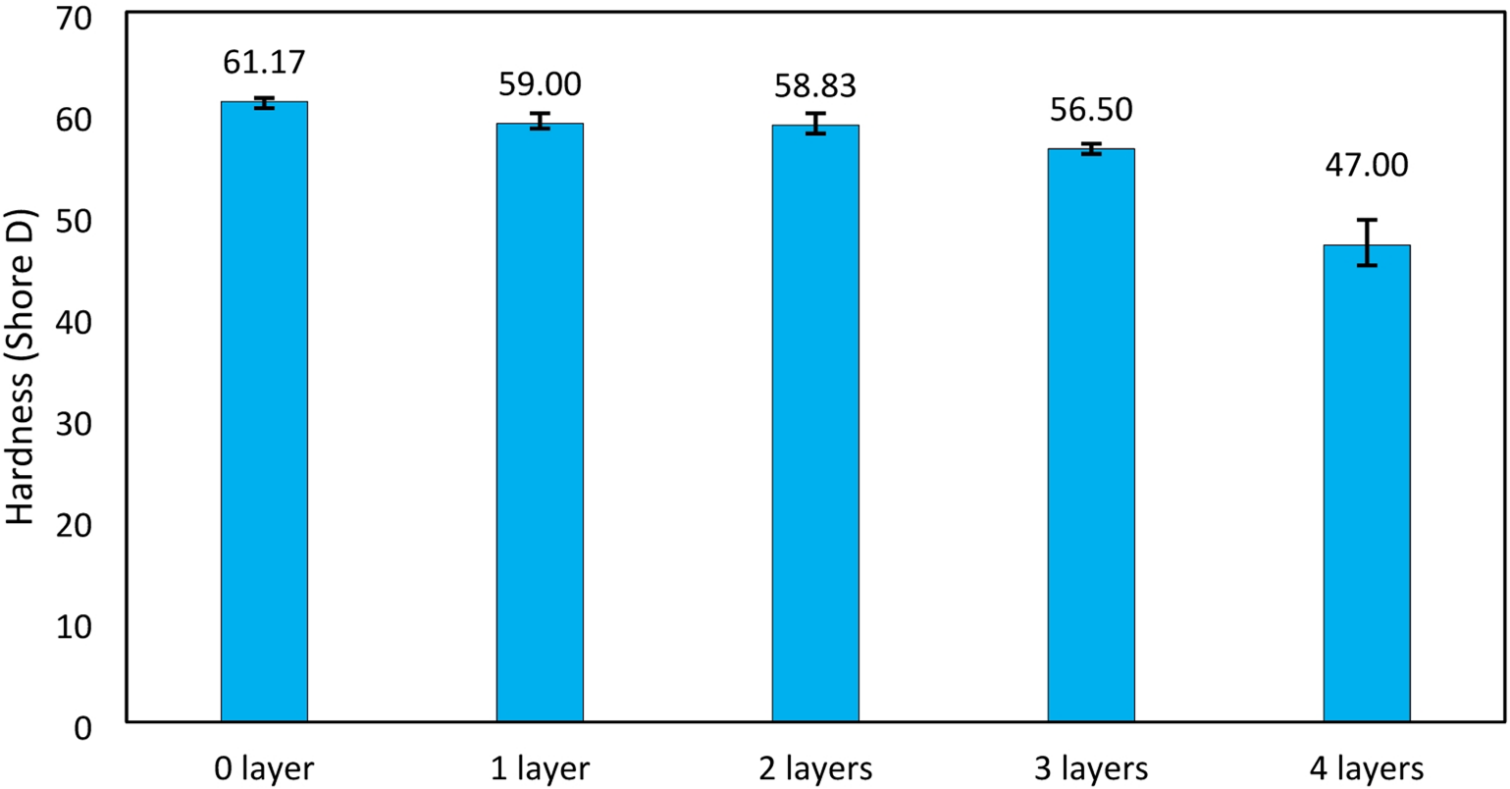
Hardness value of GF-VPP composite.

In this study, it was found that as the number of glass fiber layers increased, the hardness of the composite decreased, this can be seen in Fig. 14. The hardness was used Shore D Hardness standard for hard polymers. The average hardness value of 0 layer was 61.16 HD, 1 layer was 59 HD, 2 layers was 58.83 HD, 3 layers was 56.5 HD, and 4 layers was 47 HD. This was because as the glass fiber layer increased, the sample became softer due to the dampening properties of the woven structure. Consequently, the hardness decreased as the number of glass fiber layers increased in the sample, This trend is particularly notable in VPP matrix-based composites, where the resin matrix inherently provides a rigid structure, while the addition of multiple woven glass fiber layers introduces more interfaces and flexibility, especially when interfacial bonding is not optimal. The multilayer configuration creates internal regions that can absorb or dissipate stress, reducing the overall surface resistance to indentation, which is reflected in lower hardness values. Furthermore, the decrease in hardness as the number of glass fiber layers increases is influenced by the non-homogeneous distribution of stress within the laminate. The inner layers may not be fully impregnated or bonded with the matrix, especially in VPP-based systems, leading to localized soft zones. This non-uniform microstructure allows the indenter to penetrate more easily during hardness testing. This phenomenon was also reported in previous studies^59^.

Hardness in polymer composites is often correlated with other mechanical properties such as tensile strength and flexural strength. In this study, a similar trend was observed: as the number of glass fiber layers increased, both the tensile and flexural strength of the composite decreased. This suggests that the reduction in hardness may be an indicator of poor fiber-matrix adhesion and inadequate stress transfer across the layers, which also compromises the composite’s performance under tensile and bending loads. Therefore, hardness measurements not only reflect surface resistance but can also give insight into the internal structural integrity of the multilayer composite.

### Density test results

**Fig. 15:**
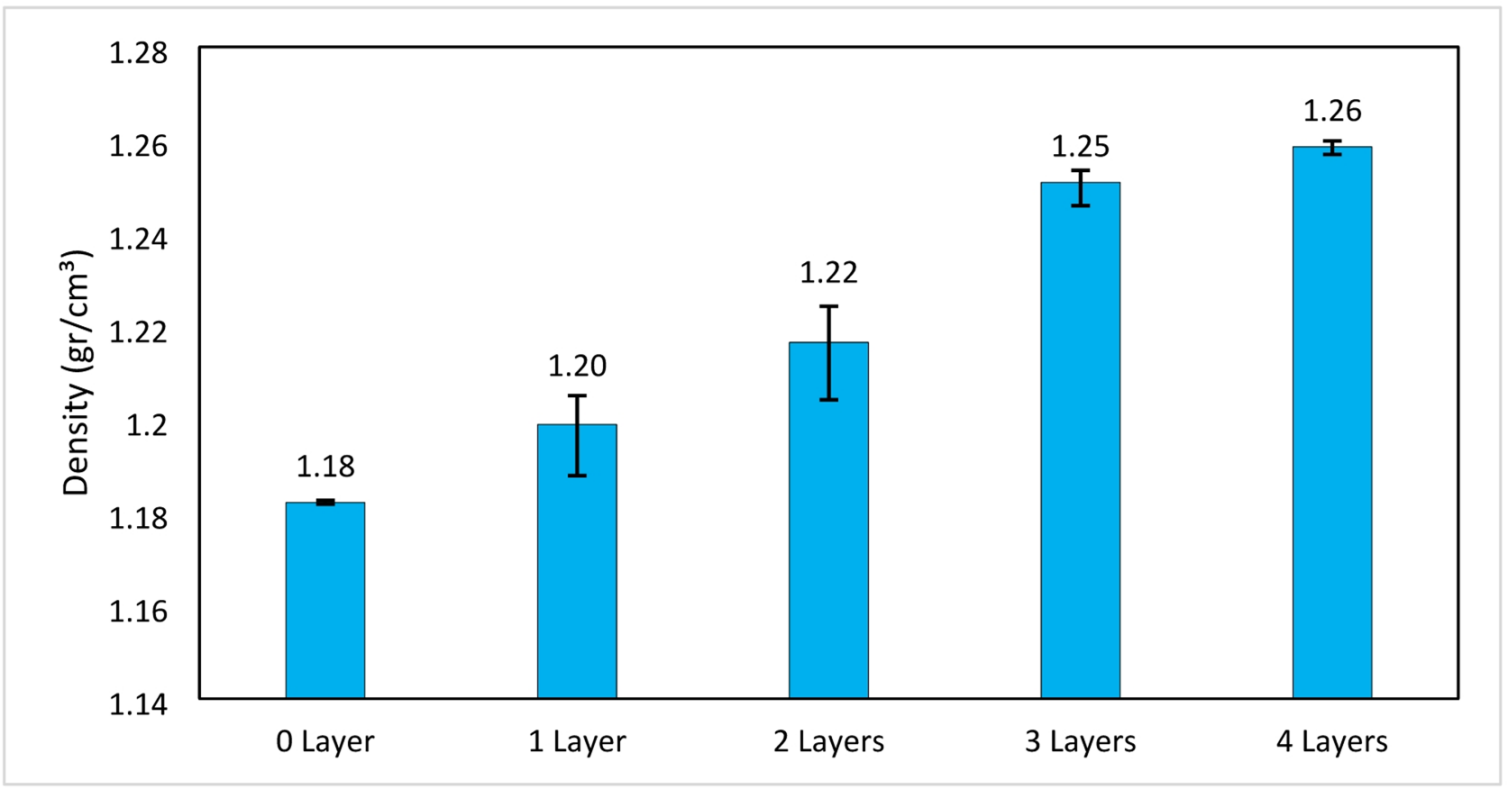
Density test results of GF-VPP composite.

In this study, the increasing number of glass fiber layers increased the density of the specimen, composites with higher fiber content and lower porosity tend to have higher densities^60,61^. However, this needs to be explored further, especially on the effect of porosity due to voids, as well as the effect of resin infiltration into the GF microstructure. The density of the specimen can be seen in Fig. 15. Specimens with 0 layers of glass fiber variation had an average density of 1.18 g/cm^3^, those with 1 layer had an average density of 1.19 g/cm^3^, those with 2 layers had an average density of 1.21 g/cm^3^, those with 3 layers had an average density of 1.25 g/cm^3^, and those with 4 layers had an average density of 1.258 g/cm^3^. The density of the specimens increased as the number of glass fiber layers increased. This indicated that glass fiber had a higher density than VPP resin, resulting in a higher overall composite density as more layers were added. The increase in density was generally correlated with an improvement in tensile strength and stiffness, as more fiber meant more material to bear the load. However, the increase in density also contributed to a reduction in flexibility and a higher likelihood of delamination, especially if the adhesion between layers was not optimal. This was observed in this study, as evidenced by the bending test results, which decreased with the addition of glass fiber layers in the VPP resin matrix. Calculations of theoretical density, relative density, and void content for GF-reinforced 3D stereolithography composite specimens can be used to prove this assumption, where the calculation results can be seen in Table 2. This calculation uses eSUN Standard resin matrix density data of 1.13 g/cm^3^, while GF has a density of 1.64017 g/cm^3^ and a consolidated thickness of 0.2 mm. Composite porosity can be calculated from the air content trapped within the material structure, calculated by the formula Void $\:\text{C}\text{o}\text{n}\text{t}\text{e}\text{n}\text{t}\:\left(\text{\%}\right)\:=\:\left(\frac{{\uprho\:}\:\text{t}\text{h}\text{e}\text{o}\text{r}\text{e}\text{t}\text{i}\text{c}\text{a}\text{l}}{{\uprho\:}\:\text{r}\text{e}\text{a}\text{l}\:}\right)\times\:100$.

**Table 2:** Theoretical density, relative density, and void content calculation results.

GF Layer	Thickness (mm)	Total Volume (cm^3^)	GF Mass (g)	GF Volume (cm^3^)	Resin Volume (cm^3^)	Resin Mass (g)	Total Mass (g)	ρ theoretical (g/cm^3^)	ρ real (g/cm^3^)	% Relative Density	% Void Content
0 layer	3.86	5.79	0	0	5.790	6.543	6.543	1.130	1.1821	104.61%	-4.61062
1 layer	3.643	5.4645	0.49	0.30	5.165	5.836	6.328	1.225	1.1989	97.85%	2.153
2 layers	3.953	5.9295	0.98	0.60	5.330	6.022	7.006	1.315	1.2165	92.53%	7.466
3 layers	4.136	6.204	1.48	0.90	5.304	5.994	7.470	1.408	1.2509	88.82%	11.177
4 layers	4.309	6.4635	1.97	1.20	5.264	5.948	7.916	1.504	1.2585	83.68%	16.319

In the pure VPP material (0 GF layer), the void content is negative at -4.611%, indicating that the actual density of the molded material is higher than the theoretical density. Theoretically, pure composites without fibers should have a homogeneous and void-free structure, or a small void content, because there is no interphase interference as in the fiber reinforcement system, as evidenced by the void content of VPP without GF reinforcement in the SEM analysis of Fig. 8. In the VPP process, the photopolymer resin undergoes gradual polymerization through layer-by-layer exposure by UV or laser light. This process can cause microscopic volume shrinkage due to polymer chain incorporation (crosslinking), but at the same time, resin that has not had time to fully polymerize can also infiltrate and be retained in certain areas due to the interaction of gravity and viscosity, especially at the bottom or between the molds. Thus, the final mass of an VPP mold can be slightly higher than its theoretical mass, while the volume remains fixed, which ultimately results in a greater real density, giving rise to a negative void content.

In the VPP composite material with 1 layer of GF, the void content value increases to 2.153%. This increase in void content value can occur due to the liquid resin not being able to perfectly penetrate into the microscopic gap structure of the fiber, there are areas where the GF microscopic gap, especially at the bottom, is not well impregnated with resin and is not filled with resin, as evidenced by microscopic observations in Fig. 13., this is what gives rise to the potential porosity of the GF-reinforced VPP 3D composite structure because the microscopic gap of this fiber has the opportunity to make air trapped in the microscopic gap. This phenomenon also occurs in composites with 2-layer, 3-layer and 4-layer GF reinforcement, where the more layers of GF, the more areas of GF microscopic gaps that are not impregnated with resin, as evidenced by the void content that continues to increase as the GF layer in the composite increases, and it can be seen that the void content value jumps significantly to 16.319% in composites with 4 layers of GF. High porosity can negatively impact the mechanical properties of the material, such as reducing tensile strength, compressive strength and resistance to cyclic loading, as voids within the material can act as initiation points for cracks or stress concentration zones.

The additional GF layers in the composite structure correspondingly caused a progressive decrease in flexural strength, from 28.52 MPa at 0 layers (no reinforcement) to 17.59 MPa at 4 GF layers. This phenomenon is also in line with the increase in void content in the composite structure which also increases with the number of GF layers, from − 4.611% at 0 GF layers to a significant jump reaching 16.319% at 4 GF layers, this can be seen in Table 1. Void content in composites, especially in the interface area between matrix and GF fiber reinforcement, can be a structural weak point due to the lack of adhesion force and mechanical interlocking area between resin and GF. At 0 layers of GF, the flexural strength reaches the highest value because the material is fully homogeneous, void-free, and the resin bonds cohesively to the maximum without interface interference. After the addition of 1 and 2 layers of GF, the flexural strength decreased sharply to 22.66 MPa and 22.64 MPa, this occurred due to the formation of deep porosity in the composite structure which was expressed by the void content value increasing to 2.153% and 7.466%. In the VPP composite with 3 layers of GF, the void content value reached 11.177%, and the flexural strength consistently showed a decrease reaching 20.55 MPa. This condition worsened at 4 layers of GF, where the void content reached the highest value of 16.319%, while the flexural strength touched the lowest value at 17.59 MPa. This proves that there are areas in the microscopic gap that are not well impregnated with resin, especially at the bottom of the GF layer structure, causing weak resin-matrix interface bonding which ultimately leads to a decrease in the flexural strength of the composite material.

### Digital image correlation (DIC) validation results

In this study, the Digital Image Correlation (DIC) method was implemented through a series of structured steps using MATLAB and Ncorr software. The process began with the preparation of the specimen surface, which was painted with a black speckle pattern on a white background to facilitate accurate motion tracking. The specimen was then recorded using a Canon 750D camera mounted on a stabilized tripod, with settings such as exposure and aperture adjusted according to experimental needs. The recorded video was subsequently converted into individual frames using ImageJ software, capturing the sequence from the initial undeformed state to the point of fracture or deformation. The first step in MATLAB processing was uploading the reference image, selected from the frame showing the specimen in its undeformed condition. Image preprocessing followed, including grayscale conversion, contrast enhancement, and noise reduction to optimize feature detection. The Region of Interest (ROI) was then defined to focus the analysis on critical specimen areas.

**Fig. 16:**
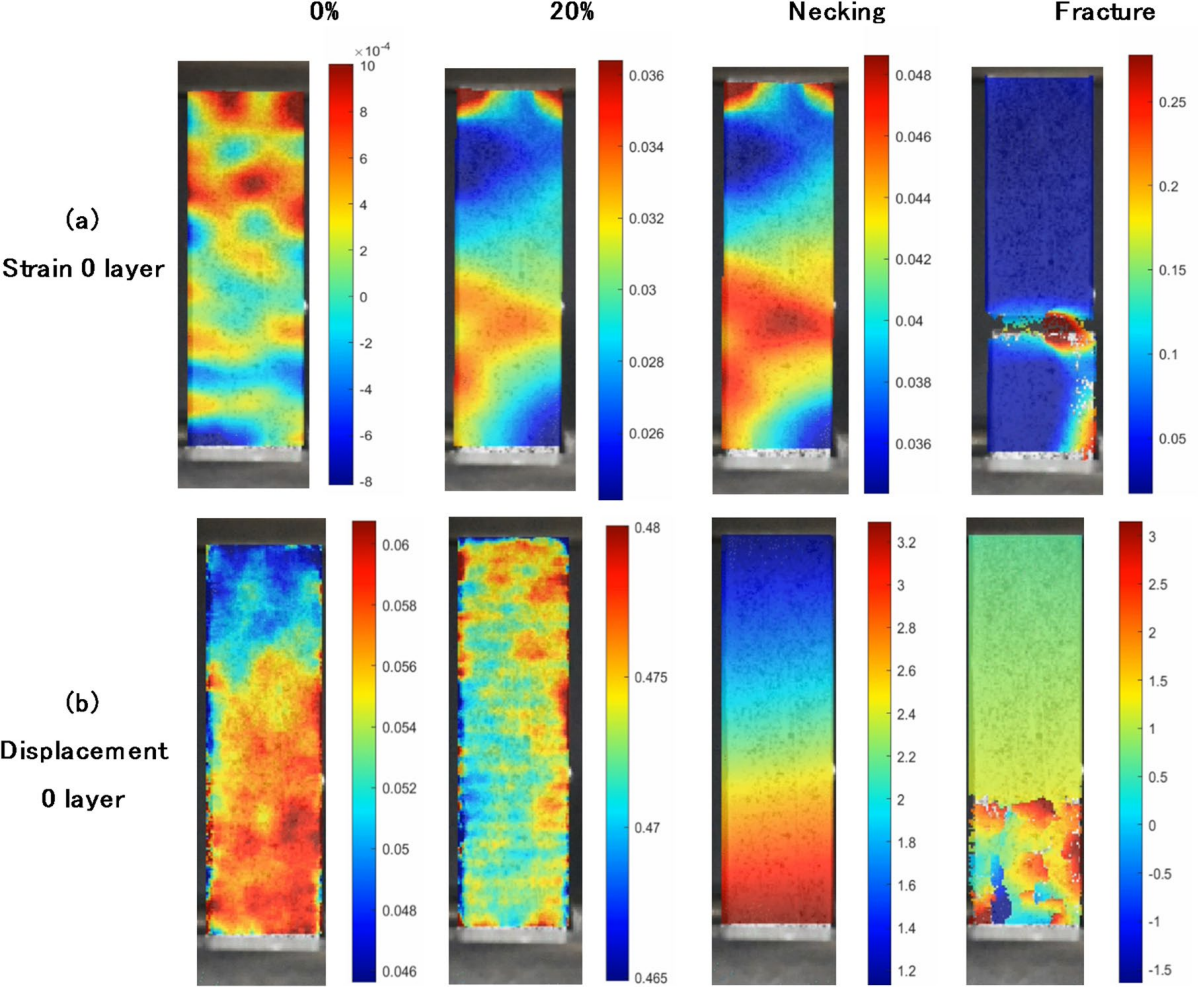
Results of analysis of 0 layer specimens with DIC: (**a**) Strain distribution, (**b**) Displacement distribution.

The DIC algorithm analyzed the speckle pattern by dividing the image into smaller subsets and tracking their movement through numerical optimization, comparing the reference image with deformed frames to compute displacement and strain fields. Region and seed parameters were initialized, and subset radius and spacing were adjusted to balance resolution and computational load. Calibration was conducted to convert pixel data into real-world units (mm), based on a specimen width of 15 mm. Strain radius was also optimized, with a value of 13 used based on Ncorr guidelines. For the tensile test on a specimen without fiberglass layers (0-layer GF), the DIC results yielded a strain value of 0.0647 and a displacement of 3.3635 mm, as shown in Fig. 16a, b. These values were compared against experimental data to assess the accuracy of the DIC method. Any discrepancies observed were attributed to factors such as lighting conditions, speckle pattern quality, or image resolution. Overall, the DIC analysis provided valuable insights into deformation behavior, strain localization, and the mechanical performance of the tested material.

Based on visual analysis of the strain map in Fig. 16a, the Digital Image Correlation (DIC) results show a clearly visible grid pattern, particularly in the second column from the left, within a strain scale range of 0.046 to 0.06. This grid pattern appears uniform and periodically repeated, resembling a checkerboard or block segmentation pattern that commonly arises as an artifact in digital image processing. Therefore, it raises the question of whether this pattern has a strong correlation with the distribution of glass fibers (GF). Meanwhile, in other images showing higher strain levels, the pattern tends to fade or weaken, and the strain distribution appears smoother and more natural. Based on these characteristics, it is strongly suspected that the grid pattern is not the result of glass fiber distribution, but rather a numerical artifact caused by the DIC processing—specifically due to suboptimal selection of subset size and step size parameters.

**Fig. 17:**
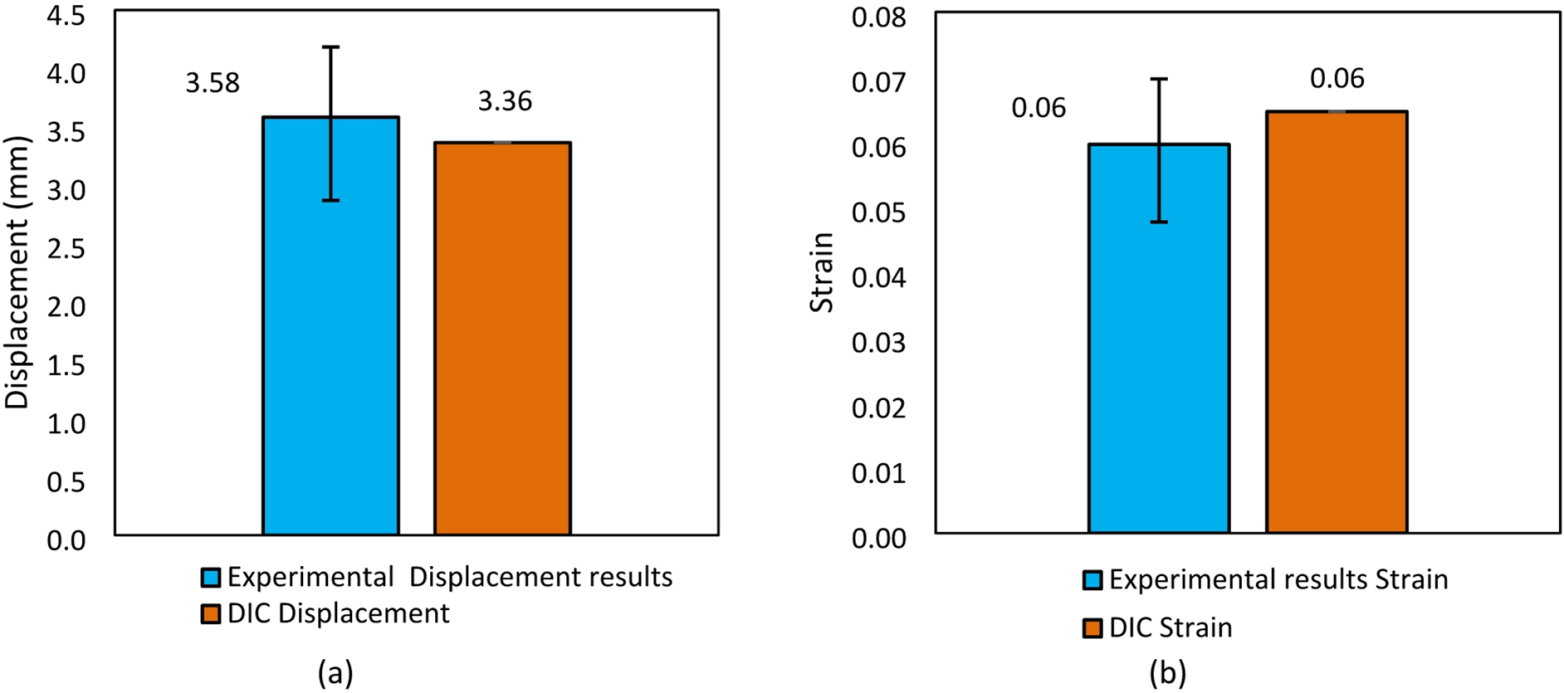
Comparison of experimental results and DIC results: (**a**) strain, (**b**) displacement.

The results of DIC were close to the experimental results. The average strain from the experimental results was 0.0597, while the average displacement was 3.5817 mm. In DIC, the strain obtained was 0.0647, and the displacement was 3.3635 mm, as can be seen in Fig. 17. This result showed that image correlation had successfully detected the movement of the speckle pattern accurately, thus it could be concluded that the experimental data obtained was valid and did not experience significant errors, either from the measuring instruments or the testing procedure. Furthermore, the slight difference between the experimental and DIC results can be attributed to minor discrepancies in the measurement techniques. The experimental method relies on physical contact sensors, which may introduce slight inaccuracies due to mechanical limitations or sensor positioning. On the other hand, DIC is a non-contact optical method that depends on image processing algorithms, which can be affected by lighting conditions, camera resolution, or speckle pattern quality.

Despite these small variations, the overall agreement between the two methods confirms the reliability of DIC in capturing strain and displacement values accurately. The DIC technique also provides additional benefits, such as the ability to visualize full-field strain distribution and detect localized strain concentrations that may not be captured by traditional contact sensors. This makes DIC a valuable tool for validating experimental results and gaining deeper insights into material deformation behavior.

### Abaqus simulation results

Tensile test simulation using Abaqus software begins with creating a part drawing according to the ASTM D3039 standard. This is followed by defining the material in the Property module, which includes the specification of ductile damage using fracture strain and displacement at failure (calculated from element length × fracture strain), density based on material variations, elastic properties such as Young’s modulus and Poisson’s ratio, and plastic properties including yield stress and plastic strain. The simulation parameters for eSun resin use a Modulus of Elasticity (E) of 2.5 GPa, while for woven Glass Fiber the Modulus of Elasticity (E) is 76 GPa. The defined material is an VPP composite with varying numbers of glass fiber layers (0–4 layers). Next, the material is assigned to a section, which is then applied to the model using the Section Assignment Manager. The following step involves meshing with a mesh size of 1 and enabling the element deletion feature to visualize specimen fracture during simulation. The loading setup involves applying a tensile force in the U2 direction (y-axis) and defining boundary conditions to act as grips by setting all degrees of freedom to zero (fixed). In the Step module, automatic stabilization is used along with incrementation settings, including a minimum increment of 1E-10, a maximum increment of 1, and a maximum number of 100,000 steps. The next step is creating a job, where the number of computer cores can be adjusted for the simulation process. Finally, in the Result and Visualization module, simulation outputs such as stress distribution and other variables are displayed, and numerical data can be extracted for further analysis and visualization in graphical form.

In this simulation, the Cohesive Zone Model (CZM) was used to represent the interface between glass fibers and the resin matrix. The CZM enables the prediction of delamination phenomena through cohesive elements or surface interactions using a traction-separation approach. The main parameters of this model include the interfacial bond strength (both normal and shear) and the initial stiffness of the interface. This approach allows for a realistic and accurate simulation of interfacial failures, such as delamination under tensile or flexural loading. To represent the stacking configuration of fiber and resin layers, a solid layered modeling approach was employed. In this method, the part is created as a three-dimensional solid (3D solid part) consisting of multiple layers representing both pure resin and resin reinforced with glass fibers. The model is constructed by dividing the part into several sections or volumes—either through geometric partitioning or by using separate parts—each of which is assigned different material properties. The unloading scenario in the tensile test simulation of GF-VPP composites is performed even though it is not shown in experiments, because it has an important value in understanding the elastic behavior of the material thoroughly. Although in experimental tensile testing the main focus is usually on obtaining the maximum strength and strain until fracture, simulation provides an opportunity to evaluate the reversible elastic response of the material when the load is released. The unloading process allows observation of the slope of the curve at load release, which is directly related to the elastic modulus and the potential for permanent deformation (plasticity or residual damage). In addition, in numerical simulations, especially using the finite element method, the unloading scenario helps verify whether the material model is linear elastic, elasto-plastic, or has modulus degradation due to inter-phase damage such as delamination or microscopic cracking. It is also useful for checking the numerical stability of the model and estimating possible hysteresis or internal energy dissipation. Thus, although unloading is not recorded in physical testing, this scenario remains technically relevant to provide a more complete picture of the mechanical behavior of composite materials, as well as a means of validating the simulation model against the material property assumptions used.

**Fig. 18:**
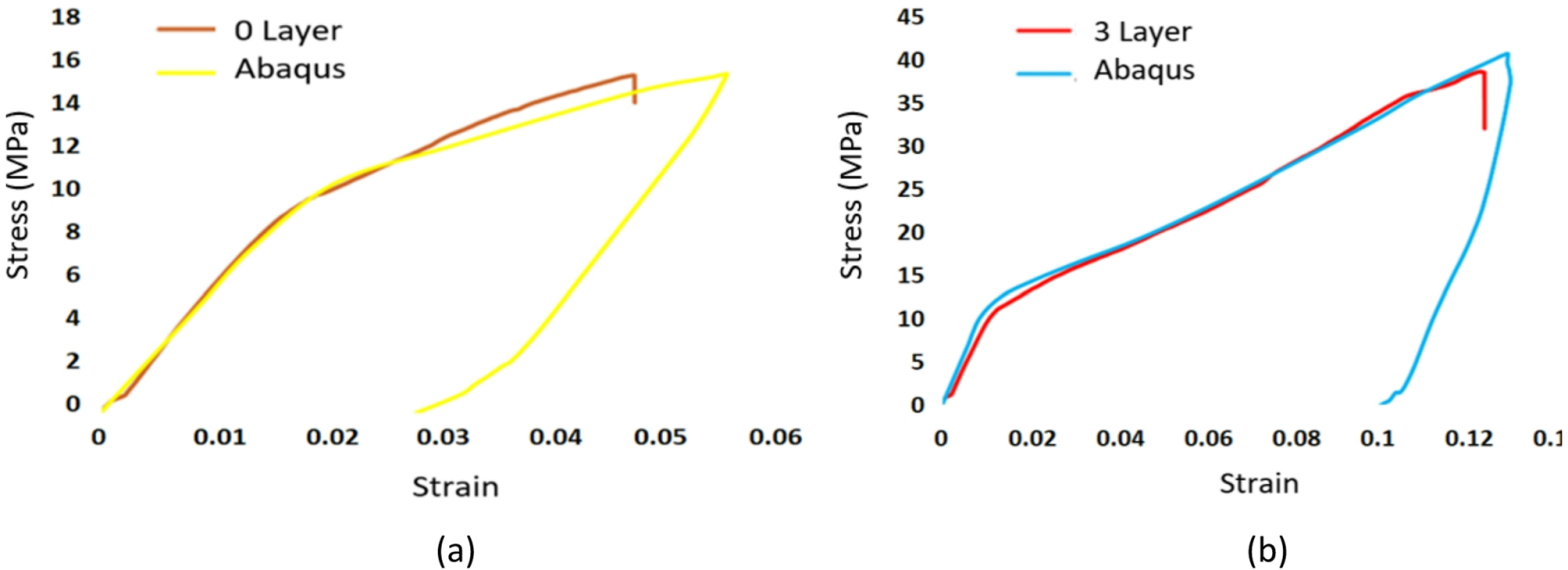
Comparison of experimental stress-strain curves with Abaqus simulation results: (**a**) 0 layer, (**b**) 3 layers.

The results of the tensile test simulation using finite element analysis (FEA) with Abaqus software were compared with experimental data. The data collected and analyzed were stress-strain curves. The comparison of stress-strain curves between the Abaqus FEA simulation and the experimental results showed minor differences, particularly in the plastic region. However, on average, both the simulation and the experiment exhibited the same ultimate tensile strength (UTS) for the compared specimens. This can be seen in Fig. 18, specifically in the comparison of numerical simulation results and real experiments for specimens with 0-layer and 3-layer variations.

**Fig. 19:**
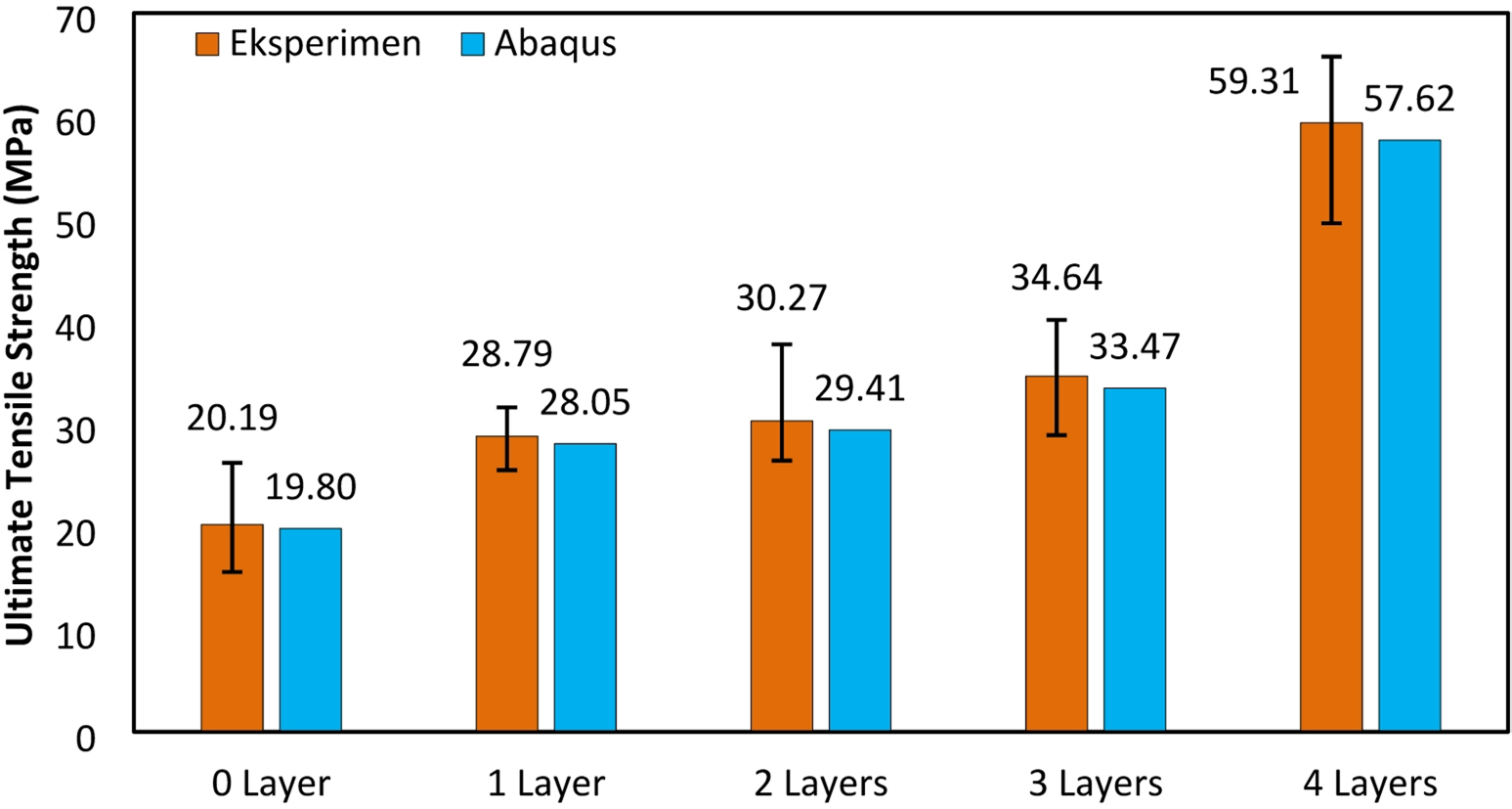
Comparison of Abaqus & experimental ultimate tensile strength.

**Fig. 20:**
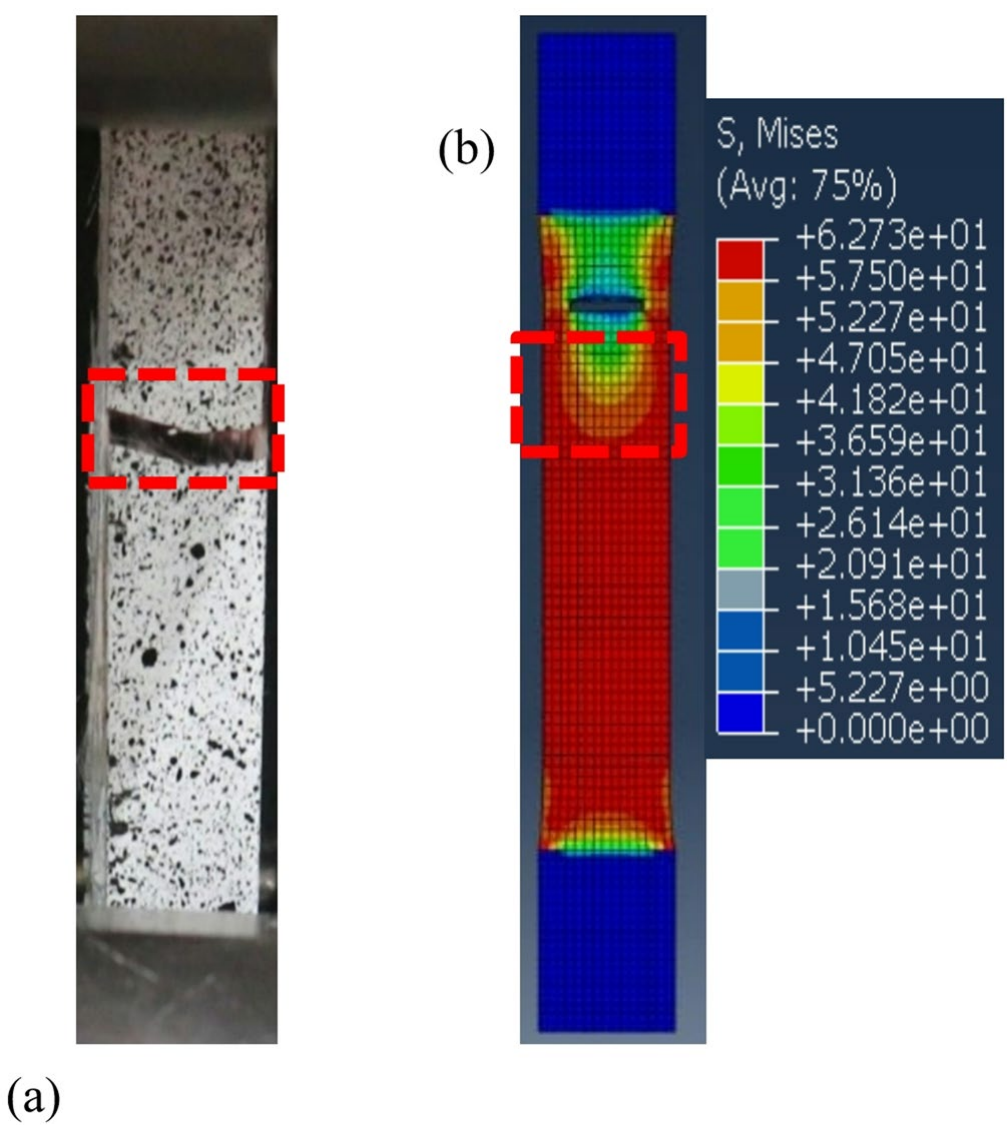
Failure mode in the tensile test of GF-VPP: (**a**) 4 layers experimental, (**b**) 4 layers Numerical simulation.

In the FEA simulation using Abaqus, the average ultimate tensile strength (UTS) was obtained for each layer. The UTS for 0 layers was 19.8 MPa, for 1 layer 28.05 MPa, for 2 layers 29.40 MPa, for 3 layers 33.46 MPa, and for 4 layers 57.61 MPa. The UTS results from the simulation were close to the experimental values, validating the modeling approach in Abaqus. The simulation used data from all experimental specimens, and the average value was taken. This ensured the numerical analysis closely followed actual conditions, improving reliability. The minimal discrepancy between simulation and experiment suggests the material properties and boundary conditions were well-calibrated. The comparison in Fig. 19, shows that increasing glass fiber layers improved UTS, confirming its reinforcing effect. The close agreement between simulation and experimental data highlights the Finite Element Method (FEM) as an effective tool for predicting composite material properties and optimizing performance. The load or stress distribution from the Abaqus simulation on the tensile test specimens illustrates the regions with the highest stress distribution, which have a higher probability of failure. The failure areas of the tensile test specimens in this simulation closely resemble the failure regions observed in real testing^62,63^, for further analysis, FEA can also be used to analyze damage mechanisms^64^. The failure patterns in both the numerical simulation and real experiments, 4 layers variations can be seen in Fig. 20.

The tensile test results for VPP composite material reinforced with 4 layers of fiberglass have shown that the fracture pattern in the tested specimen and the simulation results using the Finite Element Analysis (FEA) method with Abaqus software exhibit significant similarity. This strong correlation indicates that the numerical model used in the simulation has effectively captured the mechanical response of the material under tensile loading. The consistency between the experimental and simulated fracture patterns suggests that critical parameters, such as material properties, boundary conditions, and meshing strategies, have been accurately defined in the simulation. Furthermore, the ability of the FEA model to replicate the actual failure mechanism enhances confidence in its predictive capability, making it a valuable tool for analyzing and optimizing composite structures. The alignment between physical testing and computational analysis demonstrates that the modeling approach used is reliable for studying stress distribution, deformation behavior, and failure progression in fiberglass-reinforced composites.

Based on the results of the quantitative analysis, the Root Mean Square Error (RMSE) values for the Digital Image Correlation (DIC) analysis of displacement and strain parameters showed error levels of 4.75% and 3.86%, respectively. All of these values fall below the 5% threshold, which is generally accepted in the validation of experimental and numerical methods. Meanwhile, the comparison between Finite Element Analysis (FEA) simulation data (Abaqus) and experimental results showed a relative error of 3.07% compared to the average experimental tensile strength value of 34.64 MPa. This low error indicates that the numerical model used has a high level of accuracy and is valid in predicting the mechanical behavior of the tested material. The Digital Image Correlation (DIC) method used in experimental measurements demonstrates high accuracy and strong consistency with the numerical predictions from Finite Element Analysis (FEA). In other words, the strong correlation between DIC and FEA not only validates the reliability of the applied simulation method but also reinforces the credibility of the experimental approach in measuring deformation and mechanical responses of composite materials. This high level of accuracy provides a solid foundation for the use of DIC and FEA as complementary tools in experimental studies and the development of advanced material designs based on simulations.

### Limitations and future work

As a mitigation strategy for potential interfacial failure, we recommend several approaches that can be applied in future studies. Future work should focus on optimizing resin formulations and fiber surface treatments to enhance adhesion and flexural strength while minimizing the risk of delamination. Surface optimization can be achieved through techniques such as sandblasting or plasma treatment applied to the matrix layer or glass fibers, which enhance both mechanical and chemical bonding. Resin modification, particularly the use of coupling agents like silane, can further improve interfacial adhesion between the matrix and the fibers. Additionally, direct experimental evaluation through interfacial shear strength tests (e.g., ASTM D3165 or ASTM D5868) is recommended to objectively quantify adhesion strength across composite layers. Exploring hybrid reinforcements—such as combining glass fiber (GF) with carbon fiber or basalt fiber—could also lead to improved mechanical performance and material reliability.

Combining glass fiber (GF) and carbon fiber (CF) in hybrid composites creates a synergistic effect that improves mechanical properties, especially flexural strength. For instance, composites with a GF/CF volume ratio of 8.8/1 showed enhanced flexural strength and pseudo-ductility, confirmed by four-point bending tests on reinforced concrete beams^65,66^. Additionally, alternating GF and CF layers yielded significant gains, with one study reporting a 21% increase in flexural strength and a 40% rise in flexural modulus using a single-step compression molding process^67^. Research shows that hybrid composites reinforced with basalt and glass fibers exhibit better mechanical properties, especially flexural strength, compared to composites reinforced with only one type of fiber. The study also found that adding basalt textile layers to glass fiber-reinforced mortar significantly improves the composite’s flexural strength and deformation capacity^68,69^. This presents an opportunity for further study in the application of VPP-based composite materials reinforced with hybrid multilayer glass fiber and carbon fiber or basalt fiber.

## Conclusions

The addition of glass fiber layers in VPP manufacturing process are successfully investigated in the present study. It demonstrated that VPP resin composites reinforced with glass fiber (GF) significantly improved mechanical properties, particularly tensile strength. The best performance was observed in specimens with four GF layers, achieving an ultimate tensile strength (UTS) of 59.3 MPa, nearly three times higher than the pure resin’s 20.1 MPa. This increase in strength was attributed to the fiber layers’ ability to bear axial loads efficiently. However, the flexural test results showed a decline in flexural strength as the number of GF layers increased. This was likely due to weak adhesion between the fiber and resin matrix, leading to delamination under bending loads. The density of the composite increased with additional fiber layers, indicating that GF has a higher specific gravity than VPP resin. On the other hand, hardness values decreased as more fiber layers were introduced, likely due to the woven fiber structure dispersing applied forces more effectively. Scanning Electron Microscope (SEM) analysis of the fracture surface revealed failure mechanisms such as fiber pull-out, matrix cracking, and delamination, further supporting the conclusion that adhesion issues influenced mechanical performance. Validation using Digital Image Correlation (DIC) and Finite Element Analysis (FEA) confirmed strong alignment with experimental results, reinforcing the reliability of the findings. The Abaqus simulation results closely matched the experimental stress-strain curves, further verifying the mechanical behavior of the composites.

## Data Availability

The data are available upon request to the corresponding author.
